# Mitochondrial membrane anchored photosensitive nano-device for lipid hydroperoxides burst and inducing ferroptosis to surmount therapy-resistant cancer

**DOI:** 10.7150/thno.36283

**Published:** 2019-08-14

**Authors:** Mangmang Sang, Renjie Luo, Yidan Bai, Jun Dou, Zhongtao Zhang, Fulei Liu, Feng Feng, Jian Xu, Wenyuan Liu

**Affiliations:** 1 Department of Pharmaceutical Analysis, China Pharmaceutical University, Nanjing 210009, China; 2 Department of Natural Medicinal Chemistry, China Pharmaceutical University, Nanjing 211198, China; 3 The Joint Laboratory of Chinese Pharmaceutical University and Taian City Central Hospital, Taian City Central Hospital, Taian, 271000, China; 4 Pharmaceutical Department, Taian City Central Hospital, Taian, 271000, China; 5 Jiangsu Food & Pharmaceutical Science College, 4 Meicheng Donglu, Huaian 223003, China; 6 Hangzhou Institute of Pharmaceutical Innovation, China Pharmaceutical University, 291 Fucheng Lu, Hangzhou 310018, China

**Keywords:** Ferroptosis, lipid hydroperoxides, epithelial-to-mesenchymal transition

## Abstract

**Rationale**: Ferroptosis is a regulated process of cell death caused by iron-dependent accumulation of lipid hydroperoxides (LPO). It is sensitive to epithelial-to-mesenchymal transition (EMT) cells, a well-known therapy-resistant state of cancer. Previous studies on nanomaterials did not investigate the immense value of ferroptosis therapy (FT) in epithelial cell carcinoma during EMT. Herein, we describe an EMT-specific nanodevice for a comprehensive FT strategy involving LPO burst.

**Methods**: Mitochondrial membrane anchored oxidation/reduction response and Fenton-Reaction-Accelerable magnetic nanophotosensitizer complex self-assemblies loading sorafenib (CSO-SS-Cy7-Hex/SPION/Srfn) were constructed in this study for LPO produced to overcome the therapy-resistant state of cancer. Both *in vitro* and *in vivo* experiments were performed using breast cancer cells to investigate the anti-tumor efficacy of the complex self-assemblies.

**Results**: The nano-device enriched the tumor sites by magnetic targeting of enhanced permeability and retention effects (EPR), which were disassembled by the redox response under high levels of ROS and GSH in FT cells. Superparamagnetic iron oxide nanoparticles (SPION) released Fe^2+^ and Fe^3+^ in the acidic environment of lysosomes, and the NIR photosensitizer Cy7-Hex anchored to the mitochondrial membrane, combined sorafenib (Srfn) leading to LPO burst, which was accumulated ~18-fold of treatment group in breast cancer cells. *In vivo* pharmacodynamic test results showed that this nanodevice with small particle size and high cytotoxicity increased Srfn circulation and shortened the period of epithelial cancer treatment.

**Conclusion**: Ferroptosis therapy had a successful effect on EMT cells. These findings have great potential in the treatment of therapy-resistant epithelial cell carcinomas.

## Introduction

Almost all classical non-surgical therapies, especially chemotherapeutic which induce programmed death of cancer cells, are related to caspase-dependent apoptotic pathways 1-3. However, the overexpression of apoptosis protein inhibitors in malignant cancer cells causes poor responses for apoptotic therapies 4, 5. Moreover, current chemotherapeutic drugs loose efficacy due to the rat sarcoma virus (RAS) mutation 6-8 and multi-drug resistance (MDR) 9, 10 occurs in malignant cells to combat apoptosis. To date, cancer remains one of the leading causes of morbidity and mortality worldwide. As such, there is a continual and urgent need for the development of new types of cancer therapy. Emerging therapies include photodynamic therapy (PDT) 11-13, high intensity focused ultrasound (HIFU) 14, 15, photothermal therapy (PTT) 16, and gene therapy (GT) 17-19, immunotherapy, and magnetic hyperthermia (MHT). Although these cancer therapies are efficacious, addressing the shortcomings of classical therapies to a certain extent, they also have their limitations. For instance, HIFU and PDT are limited in their abilities to resolve metastasis and invasion of tumors. Immunotherapy requires suitable biomarkers amidst tumor heterogeneity. Therefore, more cancer therapies are needed to complement existing regimens, and to improve patient outcomes overall.

Ferroptosis is a form of regulated cell death (RCD) that occurs as a consequence of iron-dependent accumulation of lethal lipid peroxidation (LPO). This form of cell death was identified by the Stockwell laboratory in 2014 20, 21. Subsequent studies describe the distinction of ferroptosis from apoptosis, necrosis, and autophagy at the morphological, biochemical, and genetics level 20-22. Ferroptosis is initiated by the inactivation of GPX-4, an LPO scavenger, which is mediated by glutathione depletion and directly inactivates GPX-4 23. Interestingly, Viswanathan* et al* 24 found that therapy-resistant cancer cells, which cross epithelial-mesenchymal transformation (EMT), were more sensitive to ferroptosis. This meant that these therapy-resistant cancer cells were more likely to be killed by ferroptosis inducers, when compared with nonresistant cancer cells. In other words, the EMT cancer could be suppressed by ferroptosis therapy. Epithelial cancer cells, undergoing EMT processes, have the potential to metastasize and invade and be in a state of therapeutic resistance; therefore, they are difficult to treat in the clinic. The discovery of ferroptosis sensitivity mechanisms for EMT cells provides a new strategy to address this problem.

Currently, some studies have attempted to utilize a ferroptosis based nanomaterial strategy for cancer therapy. For instance, Shen* et al* 25 constructed a Fenton Reaction-Accelerable magnetic nanoparticle to cross the blood-brain barrier for ferroptosis mediated therapy of orthotropic brain tumors. The authors increased the iron supply to cancer cells by magnetic nanoparticle to induce ferroptosis. Wang* et al* 26 developed arginine-rich manganese silicate nano-bubbles to generate GSH deprived nano-bubbles to induce ferroptosis. Although these studies developed distinctive ferroptosis strategies, GSH elimination or iron supply appear unable to induce cancer-cell-specific ferroptosis. Therefore, Liu *et al* 27 established ferrous-supply-regeneration nano-engineering combined with sorafenib and a photosensitizer to enhance ferroptosis by blocking GSH/GPX-4 and increasing iron supply. This strategy combined multiple factors to induce ferroptosis which was deemed more efficient than previous studies, however photosensitizers were loaded onto nanoparticles and then diffused into cells. This not only increased the drug loading burden of nano-delivery systems, but it led to low production and availability of LPO. Importantly, the previous researchers utilized ferroptosis as a general strategy against cancer and ignored the real value of ferroptosis strategies in the treatment of EMT cancer.

To deal with these problems, we constructed a nano-device to induce a more effective ferroptosis process. In this nano-device, photosensitizers become the lipophilic ends of polymers via chemical bonding. In addition, photosensitizers were designed to anchor mitochondrial membranes after disassembly. This feature will contribute to the bursts and efficient utilization of LPO in the ferroptosis process.

Ferroptosis was characterized morphologically as that the mitochondrial membrane densities were smaller than normal mitochondria, the mitochondria crista was reduced/not present, and the outer mitochondrial membrane was ruptured 5, 28, 29. This information inferred the importance of the mitochondrial organelle in the process of ferroptosis. Mitochondria are the primary source of cellular reactive oxygen species (ROS) generation (approximately 90%) 30-32. Mitochondrial dysfunctions are closely correlated with the disruption in the balance of mitochondrial ROS 33-36. Conventional photosensitizers are often limited by their extremely short lifespan, oxygen reliance, systemic toxicity (derived from off-site localization and self-catalysis of photosensitizers), and the limited diffusion distance of ROS generated. In addition, the penetration depth of visible light activation was impacted 37, 38. Therefore, targeting the mitochondrial membrane by increasing the NIR photosensitizer penetration can perturb lipid ROS homeostasis, which is beneficial for improving the effect of ferroptotic treatment.

**Scheme 1:**
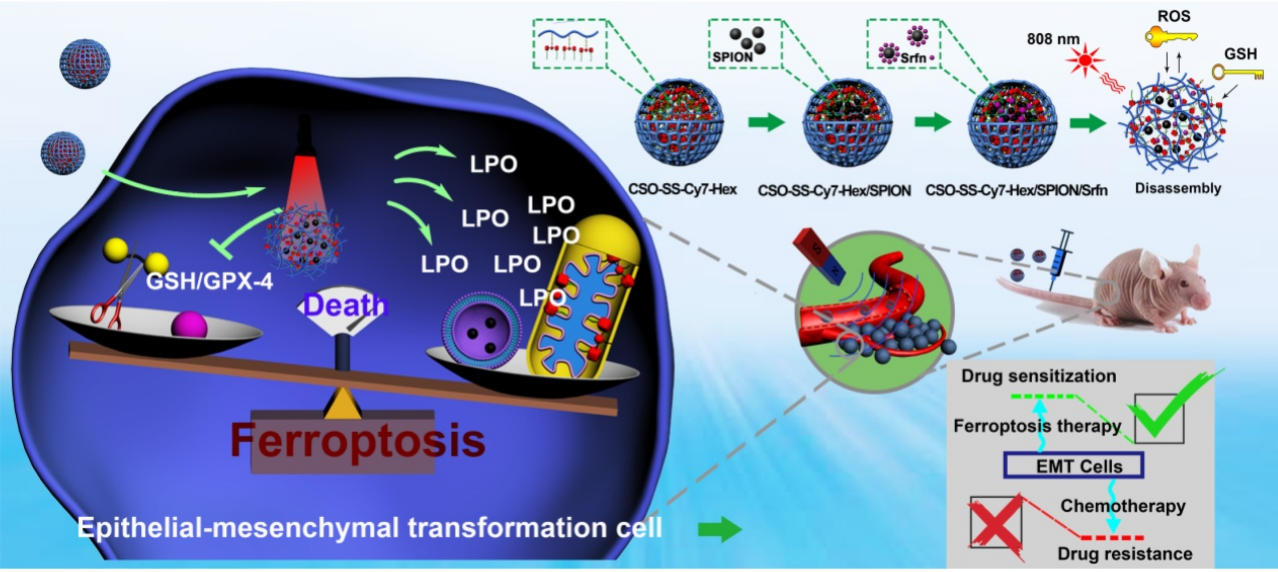
Construction of the comprehensive ferroptosis treatment strategy, which was a tumor and mitochondrial membrane targeted oxidation/reduction response, and Fenton-Reaction-Accelerable magnetic NIR nanophotosensitizer complex self-assemblies (CSO-SS-Cy7-Hex/SPION/Srfn). There are three ways to produce LPO leading to ferroptosis: (i) the oxidation/reduction response disulfide bond can consume GSH and release drug, then Sorafenib expression of system xCT, in conjunction with depression of GSH production to produce LPO; (ii) SPION (Fe^2+^, Fe^3+^) can release Fe^2+^ in late endosomes for Fenton reaction to produce LPO. (iii) the NIR nanophotosensitizer (Cy7-Hex) targets mitochondrial membranes and produces LPO under illumination.

Current research shows that there are three main indicators of ferroptosis, which are glutathione (GSH), malondialdehyde (MDA, frequently-used marker of LPO) and iron 39. We present a novel comprehensive ferroptosis treatment strategy to construct a tumor targeted and mitochondrial membrane anchored oxidation/reduction response and Fenton-Reaction-Accelerable magnetic NIR nanophotosensitizer micelles (CSO-SS-Cy7-Hex/SPION/Srfn) tumor-imaging-guided ferroptosis therapy (FT), which can consume GSH and burst lot of LPO as **Scheme 1**. The amphipathic oxidation/reduction response of the NIR nanophotosensitizer (CSO-SS-Cy7-Hex) can consume GSH and release the NIR photosensitizer Cy7-Hex, sorafenib and SPION rapidly. A large amount of ROS can be produced under illumination conditions through photosensitizers Cy7-Hex. As reported, the disulfide bonds can also be used as an oxidation-responsive linkage just like thioether bonds, which can be oxidized to hydrophilic sulfoxide or sulfone in the presence of oxidation stimuli 40. Therefore, the disulfide bond linked shell Cy7-Hex could release sorafenib and SPION rapidly in high GSH and under illumination of NIR. Cy7-Hex has two arms of hexadecane amine chain Hex linked mitochondrial targeting probe Cy7, and has a function of mitochondrial membrane-anchoring. When irradiated by the NIR laser, the assemblies could produce LPO in the mitochondria, which induced the domino effect on LPO burst 41. The overproduced LPO accumulated in the mitochondria resulted in mitochondrial collapse and irreversible cell ferroptosis. The results indicated that the mitochondrial targeting and real-time imaging of LPO burst could be achieved in living cells. SPION (Fe^2+^, Fe^3+^) can release Fe^2+^ depending on a low acid environment in late endosomes or lysosomes, and presents dramatically high catalytic activity for Fenton reaction 42. Sorafenib, expression of SLC7A11 and as GPX-4 inhibitor, leads to enhanced lipid peroxidation and onset of ferroptosis by another signaling pathway 23, 43. *In vivo* pharmacodynamic test results showed that this nano comprehensive ferroptosis delivery system, with small particle size and good biosafety credentials, can increase the circulation time of sorafenib *in vivo* and shorten the period of cancer treatment. In short, the mitochondrial membrane targeted NIR-redox response magnetic nanophotosensitizer micelles (CSO-SS-Cy7-Hex/SPION/Srfn) act on the up-, mid-, and downstream part of the signaling pathways related to ferroptosis as seen in **Figure 4A.** Moreover, we find that the CSO-SS-Cy7-Hex/SPION/Srfn complex self-assemblies mediated ferroptosis and could resist the invasion and metastasis of breast cancers during EMT. Significantly, the complex self-assemblies more easily induced cell death on mesenchymal cancer cells, a state of therapy-resistance, compared with epithelial cancer cells. These findings demonstrate great potential for clinical application on epithelial cell carcinomas. In addition to providing treatment prospects for multidrug resistant tumors, this is an effective comprehensive ferroptosis treatment strategy for the TNBC of multi-drug resistance, especially the epithelial cell carcinoma.

## Results and Discussion

### Design, Synthesis, and Characterization of Redox Dual-Responsive and Fenton-Reaction-Accelerable Magnetic NIR-Photosensitizer Complex Self-assemblies CSO-SS-Cy7-Hex/SPION/Srfn

**Figure 1:**
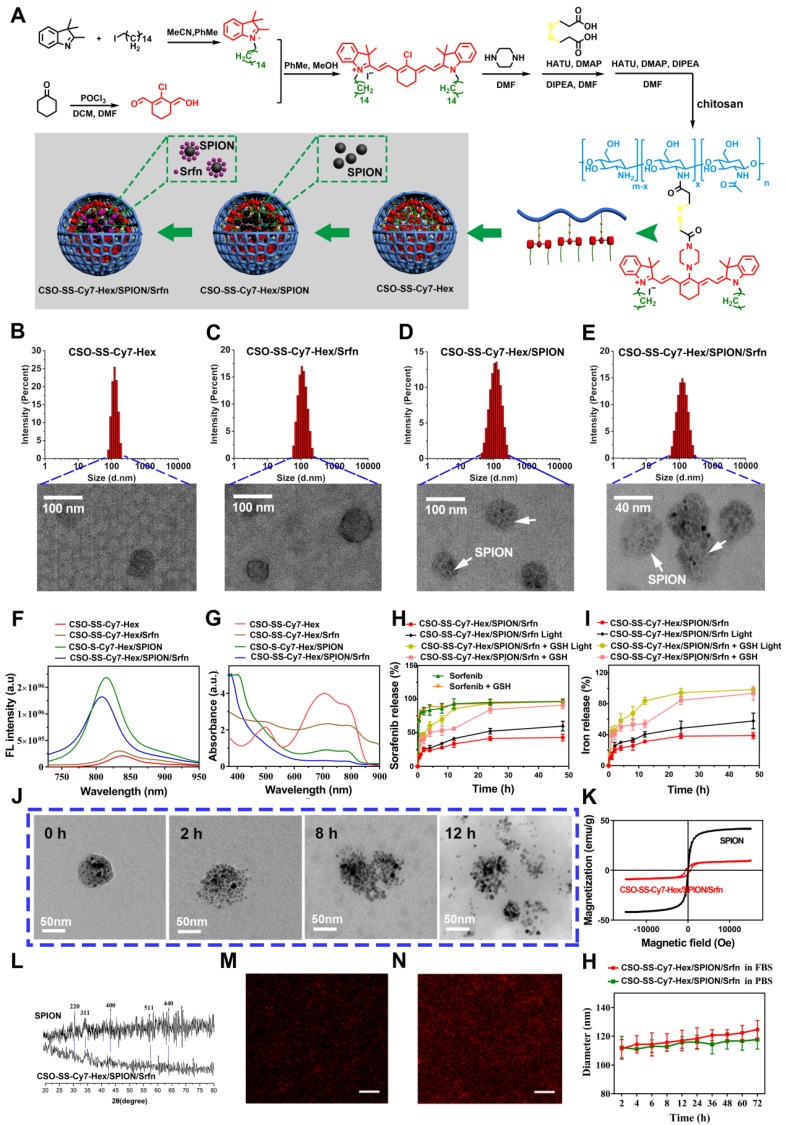
(A) Schematic illustration for the preparation of CSO-SS-Cy7-Hex/SPION/Srfn. TEM images of (B) CSO-SS-Cy7-Hex, (C) CSO-SS-Cy7-Hex/Srfn, (D) CSO-SS-Cy7-Hex/SPION, and (E) CSO-SS-Cy7-Hex/SPION/Srfn. (F) and (G) Fluorescence and absorption spectra of the CSO-SS-Cy7-Hex, CSO-SS-Cy7-Hex/SPION, CSO-SS-Cy7-Hex/Srfn, and CSO-SS-Cy7-Hex/SPION/Srfn. (H) and (I) *In vitro* release profiles of sorafenib and iron from CSO-SS-Cy7-Hex/SPION/Srfn complex self-assemblies under different simulated conditions at 37°C. The error bars in the graph represent standard deviation (n = 3). (J) Disassembly TEM images of the complex self-assemblies after incubating in 10 mM GSH for 0 h, 2 h, 8 h and 12 h. (K) and (L) Hysteresis loops and XRD pattern of SPION and CSO-SS-Cy7-Hex/SPION/Srfn complex self-assembly solution. The CLSM image (M) and (N) indicate the CSO-SS-Cy7-Hex and CSO-SS-Cy7-Hex/SPION/Srfn. Scale bar, 5 μm. (H) Stability of the CSO-SS-Cy7-Hex/SPION/Srfn in FBS and PBS at 37°C for 72 h.

The hydrophobic SPION was prepared as previously reported 44, the TEM and DLS images are shown in **Figure S11**. The results showed that the as-synthesized SPION nanoparticles were highly mono-disperse, of approximately 9 nm size. The synthesis method for the hexadecanol-dithiodipronionic acid monoester (Hex-SS-Cy7) followed the method described in the literature 45. The SPION and Srfn loaded CSO-SS-Cy7-Hex/SPION/Srfn complex self-assemblies were prepared by dialysis, as reported previously 46. **Figure 1** panel **(A)** shows the shell structure of CSO-SS-Cy7-Hex, and the scheme for the preparation of CSO-SS-Cy7-Hex/SPION/Srfn complex self-assemblies.

**Figure 1B-E** are TEM images of co-doped NPs showing well-defined complex self-assemblies of 40-80 nm in diameter. The characteristics of sorafenib and SPION loaded self-assemblies, including drug loading (DL), entrapment efficiency (EE) were summarized in **(Table S1)**. The dynamic light scattering (DLS) measurement of CSO-SS-Cy7-Hex, CSO-SS-Cy7-Hex/Srfn, CSO-SS-Cy7-Hex/SPION, and CSO-SS-Cy7-Hex/SPION/Srfn gives a hydrodynamic diameter of about 99 ± 3.25 nm, 106 ± 2.64 nm, 105 ± 1.78 nm, and 115 ± 2.23 nm with a polydispersity index (PDI) of 0.226 ± 0.08, 0.205 ± 0.12, 0.126 ± 0.09, and 0.106 ± 0.10, respectively. The size of complex self-assemblies was optimal for the EPR effect and decreased blood clearance in tumor drug delivery, consistent with previous reports 47 48. The zeta potential of complex self-assemblies was 16 mV **(Figure S12)**, which was favorable to tumor cell uptake in the tumor microenvironment 49, 50. As **Table S1** shows, the DL of sorafenib in the CSO-SS-Cy7-Hex/Srfn micelle and CSO-SS-Cy7-Hex/SPION/Srfn micelle were approximately 37.42% and 20.36% and the EE were 79% and 84%. The DL of SPION in CSO-SS-Cy7-Hex/SPION and CSO-SS-Cy7-Hex/SPION/Srfn micelles were approximately 38.53% and 27.55%, and the EE were both higher than 96%. From these results, it was clear that the loading of SPION had the potential to improve the DL and EE of sorafenib, which could be attributed to the hydrophobic fragment of the copolymer and the SPION. The ultraviolet absorption and fluorescence spectra of complex self-assemblies **(Figure 1F, G)** showed that SPION could increase the fluorescence intensity of complex self-assemblies and resulted in a blue shift in the fluorescence peak. The sorafenib release **(Figure 1H)** and iron release** (Figure 1I)** all demonstrated that illumination and high GSH were both favorable for drug release, even if high GSH plays the leading role. These results are consistent with the disulfide bond of self-assemblies resulting in a redox double response, which can release drugs rapidly under high GSH and ROS conditions 40. **(Figure 1J)** showed that the CSO-SS-Cy7-Hex/SPION/Srfn self-assembly can be easily disassembled in 10 mM GSH within 12 h, which indicated adequate reduction responses for self-assembly. The magnetic complex micelle demonstrated good superparamagnetism (**Figure 1K**), with a saturation magnetization of 9.57 emu/g, considerably less than that of SPION (45.85 emu/g) observed from the magnetization curves of SPION and CSO-SS-Cy7-Hex/SPION/Srfn complex self-assemblies. This indicated that the complex self-assemblies had an enhanced ability as tumor magnetic targets and contributed to an increased effect of EPR on the tumors. The XRD diagram (**Figure 1L**) showed that peaks at 30°, 35°, 43°, 57° and 64° corresponded to the (220), (311), (400), (511), and (440) 51, 52 phases of the face-centered cubic SPION crystal structure, indicating that the magnetite nanoparticles had good crystallinity. It also showed that the XRD pattern of CSO-SS-Cy7-Hex/SPION/Srfn complex self-assembly was consistent with SPION, which confirmed that SPION was incorporated into the self-assembly. We also demonstrated (**Figures 1M, 1N**) that the fluorescence intensity of CSO-SS-Cy7-Hex/SPION/Srfn was higher than the shell CSO-SS-Cy7-Hex, both of which were uniform. The CSO-SS-Cy7-Hex/SPION/Srfn complex self-assemblies were stable in pH 7.4 FBS and PBS at 37 ℃ for 72 h (**Figure 1H**). These results indicated that the development of these complex self-assemblies possess excellent stability and can retain their nanoscale structural integrity.

### Cellular Uptake of Complex Self-Assemblies and Magnetic Target of Cells

**Figure 2:**
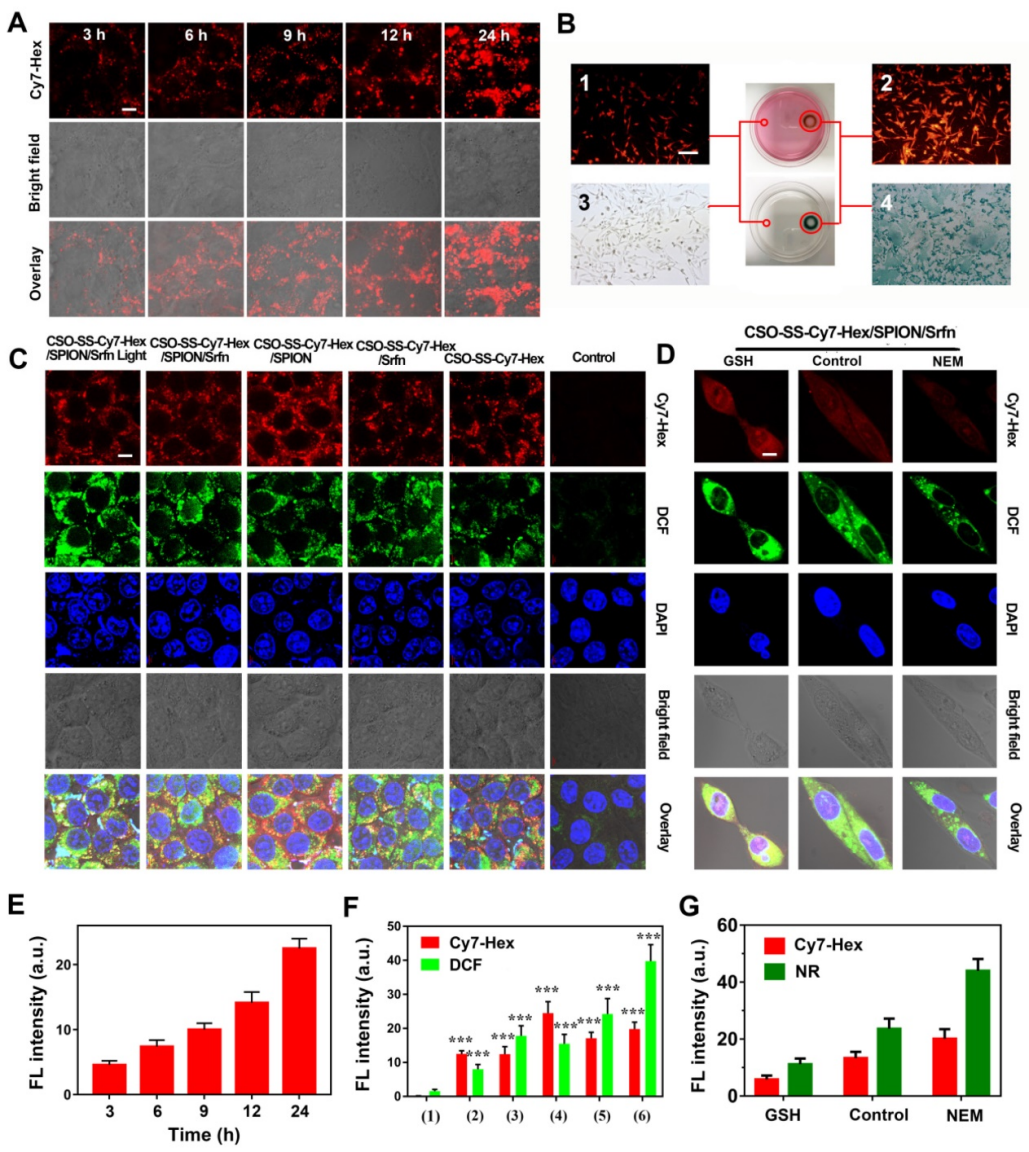
Cell uptake, DCFH-DA assay and intracellular drug release of complex self-assemblies in 4T1 and MDA-MB-231 cells. (A, E) 4T1 cells were incubated with CSO-SS-Cy7-Hex/SPION/Srfn at different times. (scale bar: 5 μm). (B) Micrographs of CSO-SS-Cy7-Hex/SPION/Srfn complex self-assemblies after 3 h incubation in an external magnetic field of MDA-MB-231 cells, (1 and 2) refer to Nile red fluorescent, (3 and 4) refer to Prussian blue staining of cells. Left and right circles indicate the control area and targeted area, respectively (scale bar: 50 μm). (C, F) DCFH-DA assay of 4T1 cells treated with CSO-SS-Cy7-Hex/SPION/Srfn Light/No Light, CSO-SS-Cy7-Hex/Srfn, CSO-SS-Cy7-Hex/SPION, CSO-SS-Cy7-Hex, and DMEM and labeled with DAPI (blue) to identify cell nuclei (scale bar: 5 μm). (D, G) MDA-MB-231 cells were incubated with NR loaded CSO-SS-Cy7-Hex/SPION/NR for 3 h after pretreatment with NEM (1 mM) and GSH (10 mM), after which cells were labeled with DAPI (blue) to identify cell nuclei. (scale bar: 5 μm).

The cellular uptake of CSO-SS-Cy7-Hex/SPION/Srfn (100 μg/mL) was monitored by fluorescence microscopy. As time increased, the signal intensity of the NIR photosensitizer Cy7-Hex increased, revealing a positive correlation of 4T1 and MDA-MB-231 cellular uptake with the incubation times **(Figures 2A, E and S13, 14)**. Flow cytometry was used to detect the cellular uptake of complex self-assemblies at different times; the signal intensity of Cy7-Hex increased as time elapsed **(Figure S15)**. An *in vitro* magnetic targeting experiment was performed to examine the magnetic targeting properties of the CSO-SS-Cy7-Hex/SPION/Srfn complex self-assemblies. A commercially available magnet (approximately 0.2 T) was placed against the outer bottom surface of the petri dish. The cells in two locations within the petri dish, referred to as the targeting area (right circle) and the control area (left circle), were investigated by Nile red (NR) fluorescence intensity **(Figures 2B1, 2B2)** and Prussian blue staining **(Figures 2B3, 2B4)**. As visualized under the microscope, both NR fluorescence and Prussian blue coloration indicated that the external magnetic field significantly increased the local concentration of the magnetic nanocarrier, implying that the magnetic nanocarrier efficiently carried cargo to the targeted area under magnetic guidance, enabling the selective and effective killing of cells in the specific area.

We evaluated the ROS level using the ROS-sensitive probe, 2′,7′-dichlorofluorescin diacetate (DCFH-DA). The order of strength and weakness of signal intensity of DCFH-DA were CSO-SS-Cy7-Hex/SPION/Srfn with Light, CSO-SS-Cy7-Hex/SPION/ Srfn, CSO-SS-Cy7-Hex/Srfn, CSO-SS-Cy7-Hex/SPION, CSO-SS-Cy7-Hex, DMEM** (Figures 2C, 2F)**. It was indicated that NIR photosensitizer shell CSO-SS-Cy7-Hex could induce ROS even without light, and after laser irradiation, ROS significant increased. The DCF intensity of sorafenib loaded micelle group was higher than SPION; the group combined CSO-SS-Cy7-Hex, sorafenib and SPION was higher than all other three groups. Flow cytometry was also used to detect ROS in cells. 4T1 cells were incubated with the 6 administration groups mentioned above for 24 h, then incubated in DCFH-DA and detected by flow cytometry. The results **(Figure S16)** were similar to the CLSM method, the ROS of CSO-SS-Cy7-Hex/SPION/Srfn with light was higher than other groups. To determine the type of ROS generated in the CSO-SS-Cy7-Hex/SPION/Srfn with light self-assemblies treated cells, hydrogen peroxide, superoxide and hydroxyl radical were detected. **Figures S17, S18, S19** indicate that after treatment with the complex self-assemblies, the increase in hydrogen peroxide was approximately 2-fold, and many hydroxyl radicals and superoxides were produced. In addition, the enhanced Fenton-reaction can promote ferroptosis, which can consume hydrogen peroxide and produce hydroxyl radicals and superoxides. Hence the increased levels of hydrogen peroxide, hydroxyl radicals and superoxides observed, and may reflect the CSO-Cy7-Hex/SPION/Srfn-inducing Fenton-reaction to produce ferroptosis. These data revealed an enhanced Fenton-reaction.

The NR loaded complex self-assemblies CSO-SS-Cy7-Hex/SPION/NR (100 μg/mL) were prepared to investigate drug release from the reduction response of the disulfide bonds of the complex self-assemblies. N-ethylmaleimide (NEM), as a scavenger for thiol 51, can react with GSH and deplete it in the cells.** Figures 2D, G,** showed that the signal intensity of NR of the GSH group was significantly stronger than the other two groups (DMEM and NEM), and the NEM group was the lowest intensity of the three groups. **Figure S20** shows that after the addition of 10 mM GSH to MDA-MB-231 cells, the complex self-assembly easily induced cell morphological changes indicating an enhanced reduction response. These results clearly illustrate the reduction response of the complex self-assemblies.

### Mitochondrial Membrane Anchoring of Complex Self-assemblies *in vitro* and *in vivo*

**Figure 3:**
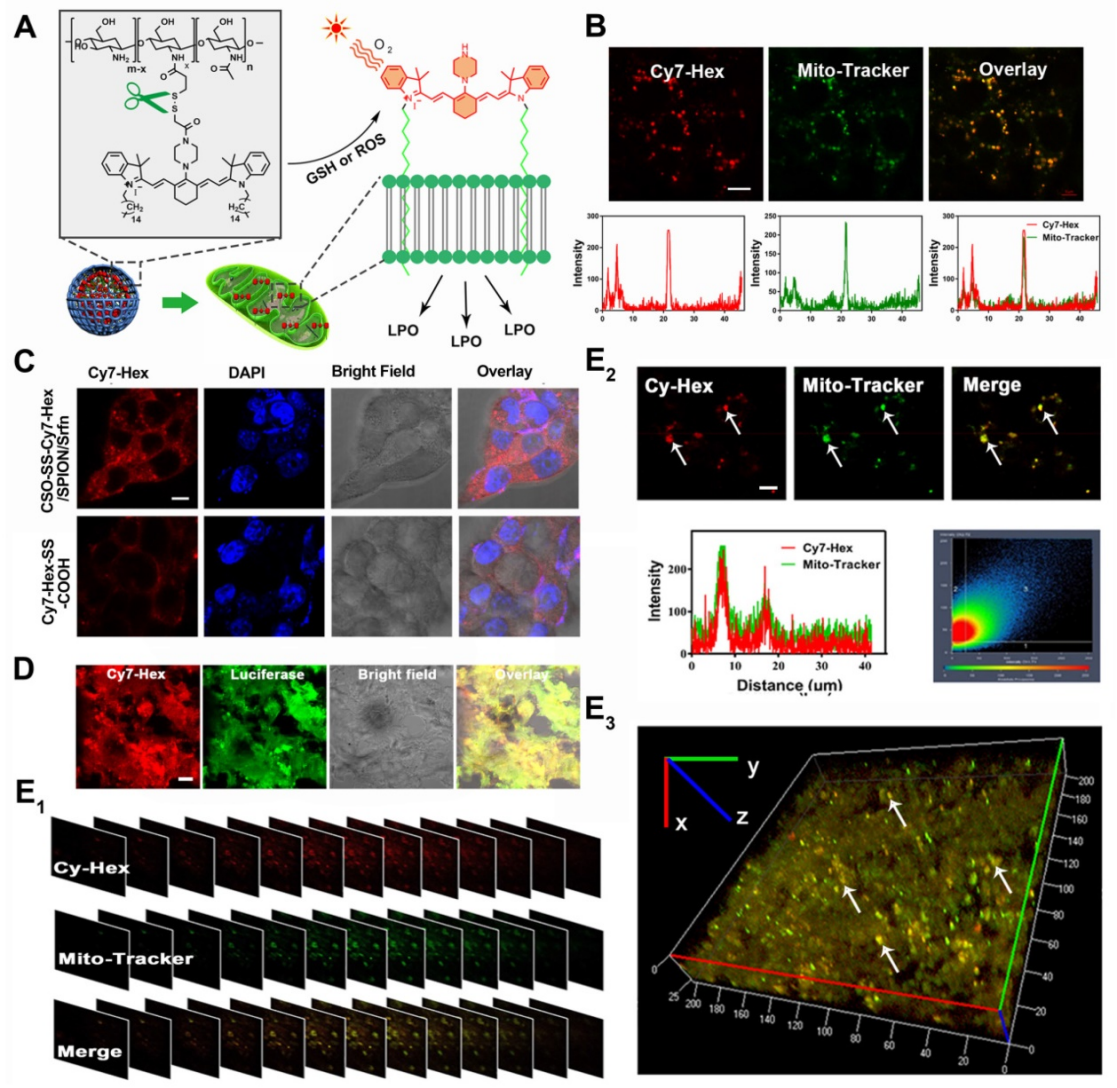
Colocalization imaging and dynamic distribution of CSO-SS-Cy7-Hex/SPION/Srfn complex self-assemblies. (A) The mechanism chart of mitochondrial membrane colocalization of CSO-SS-Cy7-Hex/SPION/Srfn. (B) CLSM images and linear profiles of 4T1 cells were used to characterize the overlap degree of mitochondrion green fluorescence of Mito-Tracker and red fluorescence of NIR photosensitizer (scale bar: 5 μm). (C) Morphological characteristics of 4T1 cells after uptake of Cy7-Hex-SS-COOH and CSO-SS-Cy7-Hex/SPION/Srfn self-assemblies and labeled with DAPI (blue) to identify cell nuclei. (scale bar: 5μm). (D) Luciferase labeled 4T1 cells bearing tumor tissue colocalization of the NIR photosensitizer. (scale bar: 5 μm). (E_1_, E_2_ and E_3_) Tumor tissue colocalization confocal magnifying scan and 3D Z-scan of tumor bearing mice. (The arrow points to the height coincidence zone. Scale bar: 5 μm).

As **Figure 3A** shows, the mechanism of mitochondrial membrane targeted complex self-assemblies is as follows: the CSO-SS-Cy7-Hex self-assembly process consists of two arms of hexadecane (Hex) chain, which can anchor to the membrane, and link with the mitochondrial targeting probe Cy7 to obtain mitochondrial membrane-anchoring ability after disassembly by high GSH and ROS 52-54.

To evaluate the capability of the oxidation/reduction response NIR nanophotosensitizer magnetic complex self-assemblies to selectively target the mitochondria in human breast cancer cell line (4T1 and MDA-MB-231), the Mito-Tracker Green (MTG) was incubated with cells after given CSO-SS-Cy7-Hex/SPION/Srfn complex self-assemblies (100 μg/mL) for 3 h. **Figures 3B and S21B** showed a high coincidence of red and green fluorescence and the overlap coefficients were 81% and 84%. **Figure 3C** shows that Cy7-Hex, which did not form self-assemblies, could target to the cell membrane; however, the self-assemblies (CSO-SS-Cy7-Hex) targeted to the mitochondria (in the cytoplasm). These results showed that the NIR nanophotosensitizer CSO-SS-Cy7-Hex could specifically localize in mitochondria membranes of breast cancer cells. **Figure S21A** shows the morphological changes of MDA-MB-231 cells at different times after the administration of complex self-assemblies. Round cells with gathered mitochondria and nuclear shrinkage were observed with increased incubation time.

We used a special 4T1 cell line, which stably expressed green fluorescent protein (GFP), to establish a visualization breast cancer xenograft tumor model. We then assessed the co-localization of Cy-Hex and GFP. As **Figure 3D** shows, the GFP almost overlapped with Cy7-Hex. This indicated that Cy7-Hex was targeted well to cancer cells in tumor tissues. And the co-localization of Cy-Hex and Mito-tracker was highlighted in tumor tissues, allowing us to conclude that Cy-Hex can be localized to the mitochondria of cancer cells. To evaluate the complex self-assemblies targeting in mitochondrial tissue, colocalization imaging experiments in 4T1 bearing tumor tissue were performed. Mito-Tracker Green (MTG) and NIR photosensitizer Cy7-Hex were employed as signals of complex self-assemblies (CSO-SS-Cy7-Hex/SPION/Srfn) in mitochondria. **Figure 3E_1_** indicated that the fluorescence of NIR nanophotosensitizer Cy7-Hex overlapped with the MTG (overlap coefficient: 0.77). This was evidenced by clear yellow signals (arrows). Furthermore, the 3D reconstitution of confocal XYZ scanning micrographs displayed the stereoscopic distribution of mitochondria in tumor tissues and also demonstrated significant overlapping with Cy7-Hex (**Figures 3E_2_, 3E_3_**). These results indicated that the complex self-assemblies were located inside tumor mitochondria and resulted in the gradual death of cancer cells.

### Pharmacodynamic Mechanism of Ferroptosis Therapy on Cancer Cells

**Figure 4:**
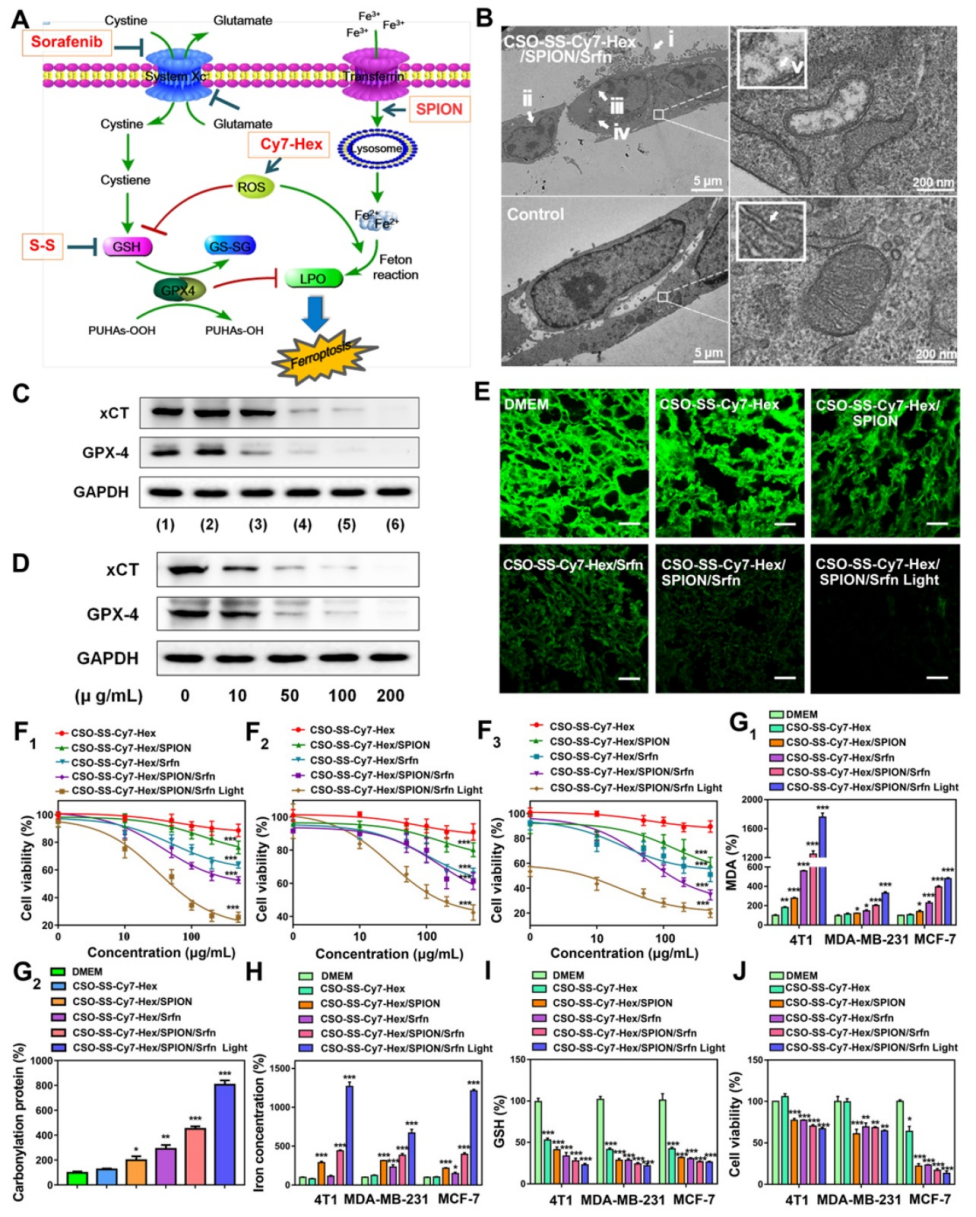
Analyses of the mechanism, efficiency, and cellular factors involved in complex self-assemblies mediated ferroptosis. (A) Schematic illustration of ferroptosis mechanism under combined treatment strategy. (B) Transmission electron microscopy of 4T1 cells treated with DMEM and CSO-SS-Cy7-Hex/SPION/Srfn self-assemblies (100 μg/mL, 3 h). (i) arrowheads, cytoplasmic and organelle swelling and plasma membrane rupture; (ii) arrowheads, nuclear atrophy; (iii) arrowheads, chromatin margination; (iv), shrunken mitochondria; (v), mitochondrial ridge decreased or disappeared. A minimum of 10^4^ cells per treatment condition were examined. (C) Western blot analysis of xCT and GPX-4 expression in 4T1 cells after the treatment with (1-6) control, CSO-SS-Cy7-Hex, CSO-SS-Cy7-Hex/SPION, CSO-SS-Cy7-Hex/Srfn, CSO-SS-Cy7-Hex/SPION/Srfn, and CSO-SS-Cy7-Hex/SPION/Srfn with light, respectively. (D) Western blot analysis of xCT and GPX-4 expression in 4T1 cells after treatment with different concentrations of the CSO-SS-Cy7-Hex/SPION/Srfn with light. (E) Immunofluorescence images of GPX-4 in 4T1 tumor tissues after treatment with different self-assemblies. (scale bar: 5 μm). (F_1_, F_2_, F_3_) Cell viability of different self-assemblies treated 4T1 cells, MCF-7 cells, and MDA-MB-231 cells, respectively. (n=6). (G_1_) Indicates knockdown 4T1 cells, MCF-7 cells and MDA-MB-231 cells were treated with different self-assemblies for 24 hours and MDA levels were assayed (n=3). (G_2_) 4T1 cells were treated with different self-assemblies for 24 hours and carbonyl protein levels were assayed (n=3). (H, I) Iron and GSH concentrations of different self-assemblies treated 4T1 cells, MCF-7 cells, and MDA-MB-231 cells. (J) Relative cell viability of different self-assemblies treated 4T1 cells after the addition of deferoxamine (DFO, 200 μM), Baicalein (10 μM) and DMEM, respectively.

Referring to the references 27, 39, we selected the biological indicators of ferroptosis, which were MDA, GSH, and iron concentration. Based on the established ferroptosis mechanism as illustrated in **Figure 4A**, sorafenib caused the inhibition of system xCT (cystine/glutamate antiporter) that typically mediates the exchange of extracellular L-cystine and intracellular L-glutamate across the cellular plasma membrane, leading to GSH depletion, GPX-4 inactivation, and the promotion of LPO resulting in ferroptosis. 55 Disulfide bond, a chemical bond that responds to glutathione reduction, can consume GSH and promote ferroptosis 56. SPION can increase intracellular iron concentration from low pH (pH 4.6) of late lysosome/endosomes, which can promote the Fenton reaction to ferroptosis 57. The NIR photosensitizer Cy7-Hex, which could target the mitochondria in human breast cancer cells, was found to produce ROS in mitochondria following irradiation by a NIR laser (**Figures 2C**, **S16)**. This combined strategy can simultaneously exert an influence on the four inducing factors of ferroptosis.

Ferroptosis is an iron-dependent non-apoptotic cell necrosis 4 and is distinct from other known forms of cell death. Ultrastructural features** (Figure 4B)** have revealed cell membrane rupture and vesicle formation, nuclear atrophy, no nuclear condensation or chromatin margination 29. We focused on the potential role of mitochondria in the ferroptosis strategy, as this organelle displays an aberrant morphology during ferroptosis. As shown in** Figure 4B**, the mitochondria seemed smaller than normal with increased membrane density and decreased or absent mitochondrial ridges. These features are similar to those observed in other studies on ferroptosis 4.

Western blot analysis was used to test the expression of xCT and GPX-4 in cancer cells. Compared with control, the administration group of CSO-SS-Cy7-Hex/Srfn, CSO-SS-Cy7-Hex/SPION/Srfn, and CSO-SS-Cy7-Hex/SPION/Srfn with light could effectively down-regulate the xCT and GPX-4 level in 4T1 cells (**Figure 4C**). It is interesting to identify that the CSO-SS-Cy7-Hex/SPION also showed the ability to downregulate the GPX-4 level. The CSO-SS-Cy7-Hex/SPION/Srfn with light group showed the best inhibition effect on the expression of xCT and GPX-4 (**Figures 4C, S22 and S23**), which might be due to laser irradiation condition, which could not only promote the ROS concentration in cells but also promoted the break of disulfide bond and increased the release of SPION and sorafenib. **Figure 4D** showed that complex self-assemblies downregulated xCT and GPX-4 levels in a concentration-dependent manner and the inhibitory effect was obvious at concentrations greater than 10 μg/mL. To investigate the expression of GPX-4 in tumor tissue, immunofluorescence was performed. **Figures 4E** and** S24** show that different groups of self-assemblies could inhibit the expression of GPX-4, and that the CSO-SS-Cy7-Hex/SPION/Srfn with light group was the strongest. These results indicated that xCT and GPX-4 played a role in the complex self-assemblies induced ferroptosis, in addition, the GPX-4 inhibition would promote the lipid peroxide level, and can be used as a significant indicator of the ferroptosis 58 59.

The 4T1 cells, MDA-MB-231 cells and MCF-7 cells were treated with various concentrations of sorafenib (**Figure S25**) and CSO-SS-Cy7-Hex, CSO-SS-Cy7-Hex/SPION, CSO-SS-Cy7-Hex/Srfn, CSO-SS-Cy7-Hex/SPION/Srfn, CSO-SS-Cy7-Hex/SPION/Srfn with light groups for 24 h, then the cell viability was measured. The cytotoxic effect of sorafenib and different self-assemblies on the cells were dose-dependent (**Figures 4F_1_, 4F_2_, 4F_3_**). The IC50 was calculated; **Table S2** shows that the maximum value of IC50 of different self-assemblies was the CSO-SS-Cy7-Hex group, and the minimum value was the CSO-SS-Cy7-Hex/SPION/Srfn with light group. The results confirmed that the ability of comprehensive administration strategy was excellent for the induction of ferroptosis. Therefore, the CSO-SS-Cy7-Hex/SPION/Srfn assemblies developed in this study appeared to be a highly effective multifunctional nanomedicine for the treatment of cancer.

As expected, the biomarker of lipid peroxidation MDA was significantly increased following treatment with DMEM, CSO-SS-Cy7-Hex, CSO-SS-Cy7-Hex/SPION, CSO-SS-Cy7-Hex/Srfn, CSO-SS-Cy7-Hex/SPION/Srfn, and CSO-SS-Cy7-Hex/SPION/Srfn with light groups as **Figure 4G1**. In the ferroptosis process, 4-HNE and MDA react with the amino acid residues of proteins to produce carbonyl proteins 55, 60. Hence, we detected carbonyl proteins (CP) by ELISA to reflect LPO level in breast cancer cells **(Figure 4G2).** The CSO-SS-Cy7-Hex/SPION/Srfn with light group produced the highest number of CP, when compared with the other groups, which was consistent with the combined administration strategy. The level of intracellular iron as **Figure 4H** showed, CSO-SS-Cy7-Hex group was similar to the control group, the CSO-SS-Cy7-Hex/SPION group was higher than CSO-SS-Cy7-Hex/Srfn but lower than CSO-SS-Cy7-Hex/SPION/Srfn. The CSO-SS-Cy7-Hex/SPION/Srfn with light group was higher than the other five groups. The concentration of GSH decreased following treatment with the above six groups **(Figure 4I)**. These results were consistent with the combined administration strategy according to **Figure 4A**.

To further strengthen investigating the mechanism of ferroptosis, different inhibitors of ferroptosis were applied to regulate the viability of different cells (4T1, MCF-7 and MDA-MB-231 cells) under the treatment with DMEM, CSO-SS-Cy7-Hex, CSO-SS-Cy7-Hex/SPION, CSO-SS-Cy7-Hex/Srfn, CSO-SS-Cy7-Hex/SPION/Srfn, and CSO-SS-Cy7-Hex/SPION/Srfn with light groups. An iron chelating agent deferoxamine (DFO), as an inhibitor of ferroptosis, was demonstrated to significantly prevent the complex assemblies inducing cell death. Baicalein 61, a small molecule ferroptosis inhibitor, could remarkably alleviate cytotoxicity of complex self-assemblies **(Figures 4J, S26, S27)**.

To ensure the strategy is a comprehensive ferroptosis strategy rather than photothermal therapy and photodynamic therapy. We used thermal imaging and apoptosis detection to investigate the effects of photothermal therapy and photodynamic therapy after the treatment of complex self-assembly, respectively. The results (**Figure S28**) showed that the temperature of the cell suspension, which was treated with complex self-assembly, was not changed within 5 min of laser irradiation. This indicated that the photothermal efficiency of ferroptosis treatment was very low. The results of the apoptotic experiment (**Figure S29**) showed that only 1.88% of cells underwent apoptosis and 20.70% of cells underwent necrosis. These data indicated that the complex self-assembly treated breast cancer cells predominate ferroptosis (a regulated cell necrosis) 62. These results indicated that a comprehensive ferroptosis therapy in this study, rather than treatment combinations of photodynamic therapy or photothermal therapy.

### Efficacy of Ferroptosis Therapy on EMT Breast Cancer Cells

**Figure 5:**
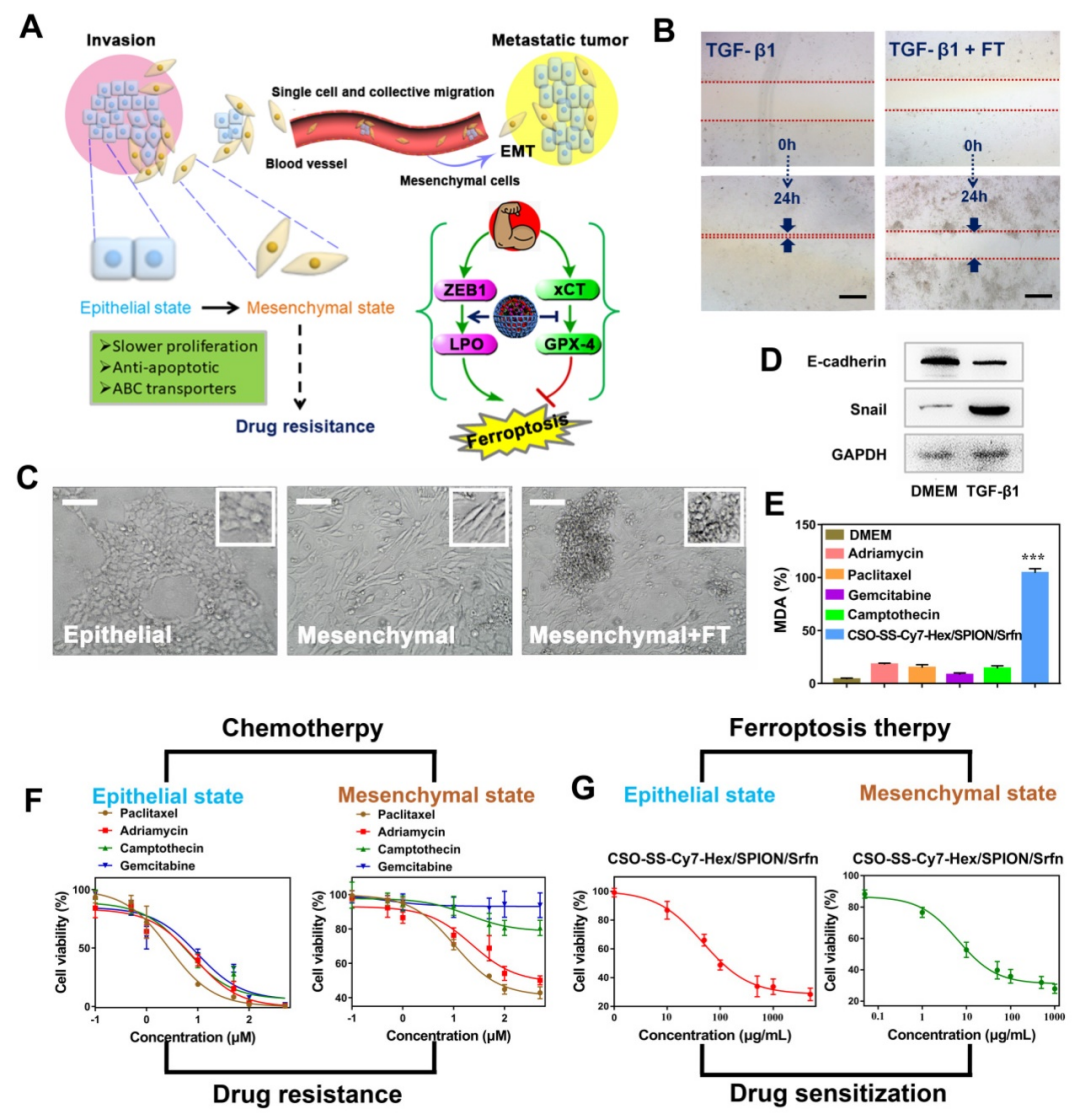
The mechanism of EMT and the efficiency of FT on EMT breast cancer cells. (A) Schematic illustration of the invasion-metastatic by EMT, which was sensitive to Ferroptosis. The scheme reference to Zhang *et al* 55. (B) *In vitro* scratch assays to test the 4T1 cells migration with or without TGF-β1 stimulation (5 ng/mL; 48 hours). The scale bar represents 200 μm. (C) Morphological changes of 4T1 cells under epithelial, mesenchymal and mesenchymal with FT. The scale bar represents 200 μm. (D) Western blot analysis of EMT markers E-cadherin and snail expression in quiescent or TGF-β1-stimulated 4T1 cells. (E) The MDA levels in TGF-β1-stimulated 4T1 cells after incubated with six groups (DMEM, Paclitaxel, Adriamycin, Gemcitabine, Camptothecin, and CSO-SS-Cy7-Hex/SPION/Srfn with light). (F) showed the cell viability after treatment with different chemotherapeutic drugs before or after TGF-β1 stimulated. (G) The cell viability after treated with complex self-assemblies before or after TGF-β1 stimulated.

The EMT program facilitates several steps of the invasion-metastatic cascade (**Figure 5A**). At the primary tumor site, induction of an EMT program allows carcinoma cells to lose cell-cell junctions, supports cancer cell dissemination in both the “single cell” and “collective migration” modes. The activation of EMT lead to the mesenchymal state cells slower proliferation rate, elevated expression of anti-apoptotic proteins, and upregulation of ATP binding cassette (ABC) transporters, which confers multidrug resistance on epithelial carcinoma 63-66.

An* in vitro* scratch assay was performed to investigate the 4T1 cell migration with TGF-β1 stimulation (5 ng/mL; 48 hours) after treatment with DMEM or complex self-assemblies (CSO-SS-Cy7-Hex/SPION/Srfn with laser) for 24 hours, the scratched monolayer was photographed at 0 and 24 hours. It was obvious showed from **Figures 5B** and **S30** that wound healing of FT group was significantly slower than control group, which was indicated that the complex self-assemblies had significant inhibition ability on cell migration. The breast cancer epithelial 4T1 cells underwent EMT after the cells were stimulated with TGF-β1 for 48 hours, during which the cells lost their epithelial honeycomb-like morphology to a spindle-like shape as **Figures 5C** and** S31** showed. Along with these morphological changes, the expression level of the adherents junction protein E-cadherin 67 was decreased, whereas the expression level of the transcription factor snail 68 was upregulated, it shows the success of EMT mode (**Figure 5D**). Interestingly, the treatment of 4T1 cells with complex self-assemblies mediated a cellular resistance to EMT, which was demonstrated in the cellular phenotypic alterations as **Figure S31** showed. The key indicator of ferroptosis was through the detection of malondialdehyde (MDA) in TGF-β1 stimulated 4T1 cells under incubated by DMEM, different chemotherapeutic drugs, and complex self-assemblies. The complex self-assemblies group with highest MDA level compared with other groups (**Figure 5E**), which made a powerful statement that the FT could mediate cellular resistance to EMT. This was consistent with the hypothesis: EMT was sensitive to ferroptosis. The cell viability was measured with or without TGF-β1 stimulated (5 ng/mL; 48 hours) 4T1 cells (**Figure 5F**), after treatment with various concentrations of Paclitaxel, Adriamycin, Gemcitabine, Camptothecin, and CSO-SS-Cy7-Hex/SPION/Srfn with light (**Figure 5F**) for 24 hours. The cytotoxic effect of different chemotherapeutic drugs on the cells was dose-dependent and had lower value of IC50 without TGF-β1 stimulated, but was found to have higher IC50 values with TGF-β1 stimulated (**Figure 5F** and** Table S4**), indicating that the cancer cells during EMT were resistance to chemotherapeutic drugs. Contrarily, the TGF-β1 stimulated cells treated with complex self-assemblies had about twice lower value of IC50 than not stimulated cells (**Figure 5G** and **Table S4**), indicating that the complex self-assemblies were sensitive to kill cancer cells during EMT. The above results indicated that the FT showed an obvious advantage to cancer treatment during EMT than CT.

### Efficacy of Ferroptosis Therapy and Biosafety Evaluation *in Vivo*

**Figure 6:**
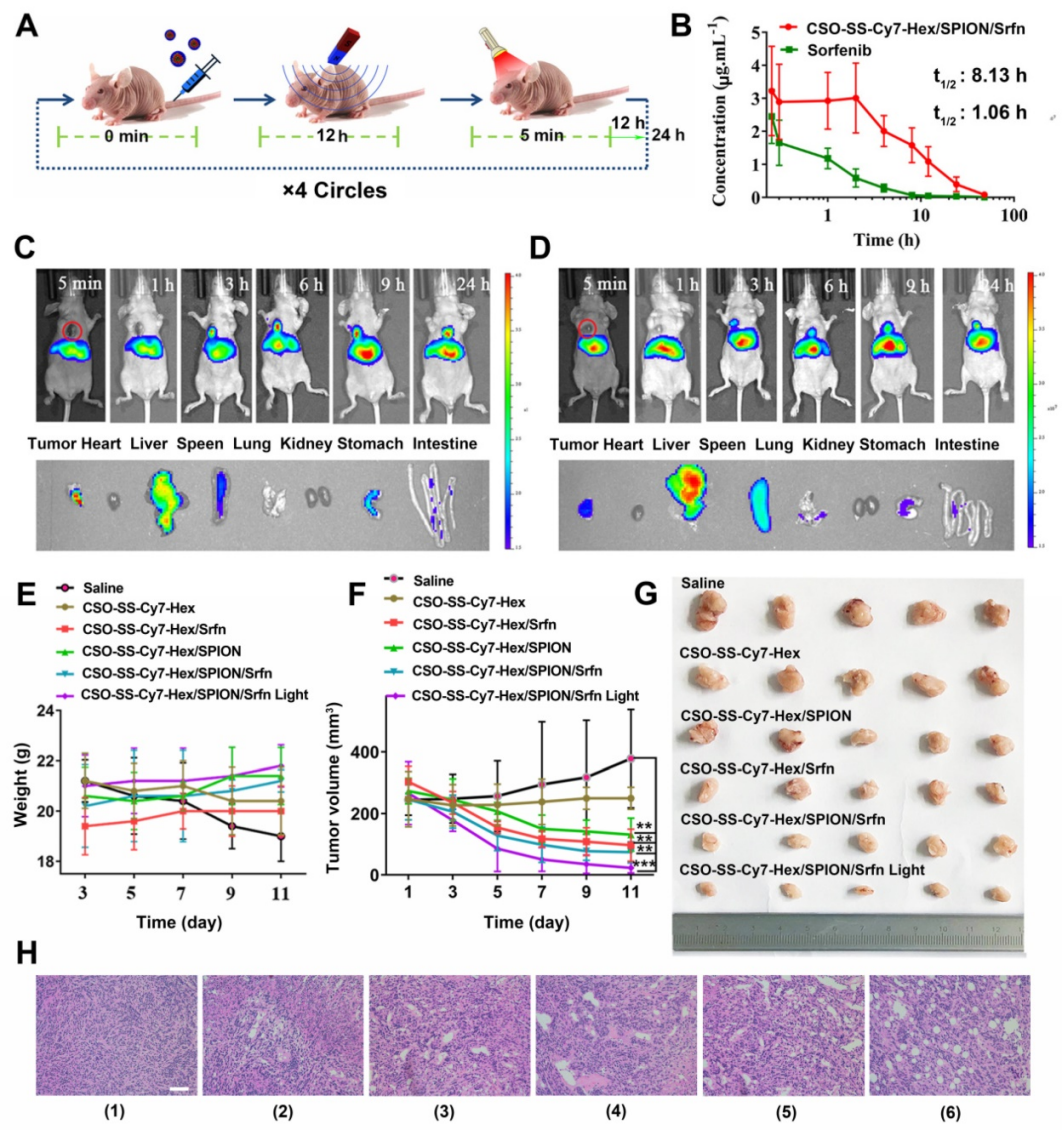
*In vivo* application of oxidation/reduction NIR nanophotosensitizer magnetic complex self-assemblies in mouse. (A) Schematic diagram of administration cycles in mice. (B) Mean blood concentration-time curve of Sprague Dawley female rats after caudal vein administration of CSO-SS-Cy7-Hex/SPION/Srfn complex self-assemblies and free sorafenib (each value represents the mean ± SD, n=6). (C and D) Showed the* in vivo* tumor magnetic targeting dynamic distribution of CSO-SS-Cy7-Hex/SPION/Srfn complex self-assemblies at different time points in tumor bearing mice monitored by the NIR fluorescence imaging system. The red ring refers to the location of the tumor. It showed the tumor exposing (MF+) (C) or not exposing (MF-) (D) to magnetic fields at 24 h, the red circle indicates an area of tumor position. *Ex vivo* fluorescence images of isolated organs (heart, liver, spleen, lung, kidney, stomach, and intestine) from the mice at 24 h after administration the complex self-assemblies. Change of mice body weight (E) and tumor volume (F) curves of six different groups (control, CSO-SS-Cy7-Hex, CSO-SS-Cy7-Hex/SPION, CSO-SS-Cy7-Hex/Srfn, CSO-SS-Cy7-Hex/SPION/Srfn, and CSO-SS-Cy7-Hex/SPION/Srfn with light) of tumor-bearing mice after FT (n=5). The irradiation power was 2.6 W/cm^2^. (G) Photographs of mice taken after treatment (n=5). (H) H&E staining of tumor tissue of six different samples as (E). (scale bar: 200 μm).

The *in vivo* therapeutic efficacies of CSO-SS-Cy7-Hex/SPION/Srfn complex self-assemblies were evaluated in tumor-bearing nude mice **(Figure 6A)**. Using wistar female rat plasma calculation peak area ratio of sorafenib and imatinib (internal standard), the analyzed concentration has a good linearity relationship at the range of 0.01-2 μg/mL with 0.999 correlation coefficient (R^2^) and the standard curve equation is calculated A=0.003C+0.052. The experiment methodology proved to be adequate for the measurement of sorafenib in plasma. After application of CSO-SS-Cy7-Hex/SPION/Srfn complex self-assemblies in rats, the plasma concentration profiles of sorafenib were showed in **Figure 6B**. The pharmacokinetic parameters were showed in **Table S3**. The t_1/2_ of complex self-assemblies loaded sorafenib had a significant enhancement when compared to free sorafenib, about 8-fold higher. The AUC_ 0-t_, AUC_ 0-U_, and MRT values of the complex self-assemblies were all significantly higher than free sorafenib. These results may be due to the instability of sorafenib and complex self-assemblies could maintain pharmacological effectivities of sorafenib for a longer time. In summary, the developed oxidation/reduction NIR nanophotosensitizer magnetic complex self-assembly system successfully improved the biocompatibility of the chemotherapeutic drug, sorafenib.

CSO-SS-Cy7-Hex/SPION/Srfn complex self-assemblies have been prepared to investigate the time-dependent bio-distribution in tumor bearing nude mice. As shown in **Figures 6C, D**, the fluorescence was sustained at the tumor location after injection from 3 h to 24 h. This indicated a tumor-specific accumulation of the drug in 24 hours after administration of the complex self-assemblies. In comparison, the fluorescence signal of exposure tumors to magnetic fields (MF^+^) was stronger than not exposure (MF^-^). These findings indicated that the magnetic treatment of the complex self-assemblies can further enhance the tumor targeting effect. The fluorescence images of the isolated different organs (heart, liver, spleen, lung, kidney, stomach, and intestine) at 24 hours showed obvious fluorescent signal at tumor, and the metabolic pathway of the complex self-assemblies was through the liver and spleen. These results indicated that the complex self-assemblies CSO-SS-Cy7-Hex/SPION/Srfn have excellent tumor accumulation ability.

Mice were randomly divided into six groups (n=5): Saline, CSO-SS-Cy7-Hex, CSO-SS-Cy7-Hex/SPION, CSO-SS-Cy7-Hex/Srfn, CSO-SS-Cy7-Hex/SPION/Srfn, and CSO-SS-Cy7-Hex/SPION/Srfn with light, an extra magnet was attached to the tumor position, when the tumor volume was about 200 mm^3^. After 11 days, there was no obvious body weight loss in all groups throughout the therapeutic course (**Figure 6E**), revealed the safety of the complex self-assemblies. The CSO-SS-Cy7-Hex/SPION/Srfn group exhibited greater suppression effect on tumor growth than CSO-SS-Cy7-Hex, CSO-SS-Cy7-Hex/SPION, and CSO-SS-Cy7-Hex/Srfn groups (**Figures 6F, 6G**). This result was consistent with the combined administration strategy of complex self-assemblies. Furthermore, the tumor growth of the CSO-SS-Cy7-Hex/SPION/Srfn with light group was most obviously among all groups (**Figures 6F, 6G**), which indicated that illumination could promote the ferroptosis treatment of complex self-assemblies. H&E staining (**Figure 6H**) was further used to investigate the tumor killing capacity and we obtain the same results. These results fully explained that the comprehensive ferroptosis system could effectively induce tumor tissue damage.

In order to investigate the biosafety of CSO-SS-Cy7-Hex/SPION/Srfn complex self-assemblies *in vivo*, the critical biomarkers BUN/CRE and ALT/AST of serum were measured to reflect the level of renal and liver damage respectively. Compared to the control group, the sorafenib group showed a high parameter of AST and BUN, which indicated that both the liver and kidney were damaged. However, it was noted that the complex self-assemblies group were not apparently damaged (**Figures S32, S33**). H&E staining **Figure S34** was further used to investigate the potential toxicity of CSO-SS-Cy7-Hex/SPION/Srfn complex self-assemblies in five organs: heart, liver, spleen, lungs, and kidney. After treatment with three groups for half a mouth, both the liver and kidney tissue cells showed obvious damage. Some cells were diffuse, and more vacuolar tissue was observed in the sorafenib group compared with other groups. This demonstrated significant renal and liver toxicity. The results confirming the caudal vein administration of CSO-SS-Cy7-Hex/SPION/Srfn complex self-assemblies was found to be bio-compatible and tolerant, indicating that the designed GA-loaded magnetic complex self-assemblies show strong biocompatibility *in vivo*.

## Conclusions

In summary, we developed a novel comprehensive ferroptosis treatment strategy to construct a tumor targeted and mitochondrial membrane anchored oxidation/reduction response and Fenton-Reaction-Accelerable magnetic NIR nanophotosensitizer complex self-assemblies (CSO-SS-Cy7-Hex/SPION/Srfn) to tumor-imaging-guided FT. This system can consume GSH and raise the concentration of LPO and iron at the same time. The cell uptake and *in vivo* tumor inhibition experiment results indicated that the complex self-assemblies demonstrated good tumor magnetic targeting and cell toxicity *in vivo* and *in vitro* by deleting the GSH by disulfide bonds, bursting ROS by illumination, and resulted in the occurrence of Fenton Reaction by increasing intracellular iron concentrations, which in turn resulted in an increase the LPO in cells. Importantly, through investigation of the EMT related experimental results, we found that the CSO-SS-Cy7-Hex/SPION/Srfn complex self-assemblies mediated ferroptosis, could resist multi-drug resistance, invasion, and metastasis of breast cancers during EMT. Our study provides a comprehensive ferroptosis treatment strategy by using oxidation/reduction response magnetic NIR nanophotosensitizer complex self-assemblies, and is potentially a very strong platform for the generation of ideas in the design of nanomedicines for the ferroptosis of multidrug resistant cancer cells. As such, these findings could contribute to the development of a highly efficient, multifunctional, and biodegradable next-generation ferroptosis inducing nanomedicine.

## Supplementary Material

Supplementary experimental section, figures and tables.
