# Supplementary material for: Mitochondrial membrane anchored photosensitive nano-device for lipid hydroperoxides burst and inducing ferroptosis to surmount therapy-resistant cancer
Source: Theranostics. 2019 Aug 14;9(21):6209–23. doi: 10.7150/thno.36283 (PMC6735518; doi:10.7150/thno.36283)
Supplement: Supplementary file 1 — Supplementary experimental section, figures and tables. [file thnov09p6209s1.pdf]

Supporting Information

Of

Mitochondrial Membrane Anchored Photosensitive nano-device for lipid hydroperoxides burst and inducing ferroptosis to surmount therapy-resistant cancer

Mangmang Sang[a], Renjie Luo[a], Yidan Bai[a], Jun Dou[a], Zhongtao Zhang[b], Fulei Liu,[c, d], Feng Feng [*, b, e], Jian Xu[*, b], Wenyuan Liu[*, a,f]

[a] *Department of Pharmaceutical Analysis, China Pharmaceutical University, Nanjing 210009, China*

[b] *Department of Natural Medicinal Chemistry, China Pharmaceutical University, Nanjing 211198, China*

[c] *The Joint Laboratory of Chinese Pharmaceutical University and Taian City Central Hospital, Taian City Central Hospital, Taian, 271000, China*

[d] *Pharmaceutical Department, Taian City Central Hospital, Taian, 271000, China*

[e] *Jiangsu Food & Pharmaceutical Science College, 4 Meicheng Donglu, Huaian 223003, China*

[f] *Hangzhou Institute of Pharmaceutical Innovation, China Pharmaceutical University, 291 Fucheng Lu, Hangzhou 310018, China*

Both WY. Liu and J. Xu are Corresponding authors.

Experimental Section

Materials Reagents and antibodies

Chitosan oligosaccharide (CSO, the average molecular weight = 5 kDa, degree of acetylation >80%) was purchased from the Dibai Chemical Reagent Co., Ltd. (Shanghai, China). 1-Hexadecanol (≥99.0%) and Phosphorus oxychloride ($POCl_3$, ≥95%) was obtained from Sinopharm Chemical Reagent Co., Ltd. (Shanghai, China); 2-(7-Azabenzotriazol-1-yl)-N,N,N',N'-tetramethyluronium hexafluorophosphate (HATU >= 98.0%), N-Ethylmaleimide (NEM, 98%) and Nile red (NR, 95%) were purchased from Macklin Inc. ((Shanghai, China); N,N-Diisopropylethylamine (DIPEA), 4-dimethylaminopyridine (DMAP, 99%) and 2,4,6-trinitrobenzenesulfonicacid (TNBS),were bought from Aladdin Industrial Corporation (Shanghai, China); Perls stain soluble kit (# G1440) were purchased from Beijing Solarbio science & technology Co.,Ltd (Beijing, China); Deferoxamine Mesylate (DFO) was obtained from Target Molecule Corp. (American); Mito-Tracker Green (# C1048), GSH and GSSG Assay Kit (# S0053), Reactive Oxygen Species Assay Kit (# S0033), Superoxide Assay Kit (# S0060); Hydrogen Peroxide Assay Kit (# S0038); Lipid Peroxidation MDA Assay Kit (# S0131) and SDS-PAGE Gel Quick Preparation Kit (# P0012AC) was purchased from Beyotime Biotechnology Co.,Ltd (Shanghai, China). Hydroxyl Free Radical assay kit (# A018) was purchased from Nanjing Jiancheng Bioengineering Institute (Nanjing, China). carbonylation proteins ELISA Kit (# JL20119) was obtained from Jianglai biology Co.,Ltd (Shanghai, China); Ultrasensitive ECL Chemiluminescence Detection Kit (# ZD310A) bought by Beijing Zoman Biotechnology Co.,Ltd (Beijing, China). Recombinant Human TGF-β1 (# 100-21) was bought from PeproTech Inc. (American); 3-(4,5-dimethylthiazol-2-yl)-2, 5 diphenyltetrazolium bromide (MTT) was ordered from the Beyotime Institute of Biotechnology (Haimen, China). Dulbecco’s modified Eagle’s medium

(DMEM), fetal bovine serum (FBS) and trypsin were purchased from the Keygen Biotech Corp., Ltd, (Nanjing, China).

Antibodies: anti-xCT/SLC7A11 (#12691) were purchased from Cell Signaling Technology (Beverly, MA, USA), Anti-Glutathione Peroxidase 4 (anti-GPX-4, # ab125066) and Goat Anti-Rabbit IgG H&L (FITC) (# ab6717) from AbCam (Cambridge, UK).

Instrument, animal and cell lines

Dynamic light scattering (Zetasizer Nano ZS, Malvern Instruments, U.K.) was used to measure the hydrodynamic diameters and ζ potentials of each product. Transmission electron microscopy (Tecnai-12 TEM (Philips Company, Holland). Fluorescence images were obtained using a confocal laser scanning microscope (CLSM, Carl Zeiss LSM700, Carl Zeiss AG, Germany). The UV−vis spectra were recorded by using an UV−vis spectrometer (UV-1900, Shimadzu). Fluorescence spectra was recorded by using an UV−vis spectrometer (RF-6000, Shimadzu). A 880 nm NIR laser (MDL−H-808-3W, Changchun New Industries Optoelectronics Technology Co., Ltd.) was used for NIR irradiation. The *in vivo* NIRF imaging was obtained using IVIS imaging systems (Perkin Elmer). The probe-type ultra sonicator (VCX-500; SONICS and MATERIALS, INC., USA).

Mouse mammary breast tumor cell line (4T1), human breast cancer cell line (MDA-MB-231) and human breast cancer cell line (MCF-7) were obtained from were obtained from the Shanghai Institutes for Biological Sciences (China) and cultured in DMEM medium contained 10% FBS at 37 ℃ in 5% CO_2 incubator. Female BALB/c nude mice and BALB/c mice (18 ± 2 g) and Sprague Dawley male rats (180-200 g) bought by Qinglongshan animal breeding farm (Nanjing, China). All animals are Specific Pathogen Free (SPF) and feeding at least one week before the experiment.

Synthesis of mitochondrial targeting oxidation/reduction response magnetic NIR-nanophotosensitizer self-assemblies (CSO-SS-Cy7-Hex).

There are 6 synthesis steps (**Figure S1)** of CSO-SS-Cy7-Hex copolymer that were as follows:

Synthesis of compound 1.

The reaction mixture of 2,3,3-trimethyl-3H-indole (1g, 6.28mmol) and 1-Iodohexadecane (2.92 g, 18.84 mmol) was stirred at 100 ℃ for 15 h in 20 mL toluene and acetonitrile mixed solvent (1:1). The mixture was added dropwise into ice diethyl ether after cooling to room temperature. The red precipitate was collected and washed with petroleum ether (30 mL). The crude product was purified by column chromatography with gradient elution using DCM and methanol (30:1, v/v) as eluent, to obtain a pale purple solid powder (1.12 g, 56.7 %).

Synthesis of compound 2.

Phosphors oxychloride (15.62 g, 101.89 mmol) in 10 mL DCM was added dropwise to a chilled solution of DMF (7.33 g, 7.7 mL, 100 mmol) in 10 mL DCM. After 30 min, cyclohexanone (5 g, 50.95 mmol) in 10 mL DCM was added into the above solution and stirred at 80 ℃ for 4 h. It was afterward cooled to room temperature and poured into ice water and kept overnight to obtain a yellow solid powder (5 g, 56.9 %).

Synthesis of compound 3.

Compound 2 (1.67 g, 5.34 mmol) and compound 2 (0.46 g, 2.67 mmol) were dissolved in 50 mL of ethyl alcohol and benzene (1:1) in a flask equipped with a Dean-Stark trap. The mixture was refluxed for 12 h to give a dark green solution. The solvent was removed under reduced pressure to obtain the crude product after the reaction was completed. The crude product was purified by column

chromatography on silica gel using DCM and methanol (40:1, v/v) as eluent, to obtain a green solid powder. (1.59 g, 89.4%).

^{1}H NMR (500 MHz, Chloroform-d) δ 8.38 (d, J = 15.0 Hz, 2H), 7.44 (t, J = 7.5 Hz, 4H), 7.29 (t, J = 7.5 Hz, 2H), 7.36 (t, J = 10.0 Hz, 2H), 6.28 (d, J = 15.0 Hz, 2H), 4.23 (t, J = 5.0 Hz, 4H), 2.78 (t, J = 5.0 Hz, 4H), 1.89 (m, 6H), 1.76 (s, 12H), 1.28 (s, 52H), 0.91 (t, J = 5.0 Hz, 3H). ^{13}C NMR (500 MHz, Chloroform-d) δ 172.31, 150.38, 144.22, 142.31, 141.11, 128.82, 127.41, 127.38, 125.33, 122.30, 110.97, 101.50, 60.42, 50.86, 49.38, 45.16, 31.95, 31.53, 30.16, 29.72, 29.71, 29.70, 29.68, 29.64, 29.59, 29.46, 29.38, 28.18, 28.04, 27.44, 27.06, 26.78. HRMS calculated for $C_{62}H_{96}ClN_2^+$ 903.72566, found 903.72634.

Synthesis of compound 4.

Compound 3 (1.05 g, 1.58 mmol) and piperazine (0.54 g, 6.30 mmol) were dissolved in 5 mL of DMF and stirred at 60 ℃ for 12 h to give a blue solution. After the reaction was completed, the solution was extracted by ethyl acetate. The solvent was removed under reduced pressure to obtain the crude product. The crude product was purified by column chromatography on silica gel using DCM and methanol (20:1, v/v) as eluent, to obtain a blue solid powder. (0.98 g, 86.8 %).

^{1}H NMR (500 MHz, Chloroform-d) δ 7.80 (d, J = 15.0 Hz, 2H), 7.43 (d, J = 10.0 Hz, 2H), 7.36 (t, J = 10.0 Hz, 2H), 7.20 (t, J = 7.5 Hz, 2H), 7.00 (d, J = 10.0 Hz, 2H), 4.08 (s, 4H), 3.93 (t, J = 10.0 Hz, 4H), 3.47 (m, 4H), 2.50 (t, J = 7.5 Hz, 4H), 1.86 (m, 6H), 1.79 (s, 12H), 1.29 (s, 52H), 0.91 (t, J = 5.0 Hz, 3H). ^{13}C NMR (500 MHz, DMSO-d6) δ172.49, 170.02, 142.46, 141.56, 140.88, 128.30, 124.05, 123.89, 122.55, 109.27, 96.17, 53.35, 50.80, 48.61, 46.27, 43.79, 31.95, 31.53, 30.16, 29.71, 29.64, 29.60, 29.55, 29.50, 29.44, 29.38, 29.34, 29.23, 29.14, 28.77, 27.24, 27.15, 26.87, 25.08, 22.71, 21.85, 14.15, 0.03. HRMS calculated for $C_{66}H_{105}N_4^+$ 953.83338, found 953.83003.

Synthesis of compound 5.

3,3'-Dithiodipropionic acid (0.33 g, 1.58 mmol) and HATU (0.60 g, 1.58 mmol) were dissolved in 5 mL of DMF, add DIPEA (0.18 g, 1.58 mmol) and stirred at 25 ℃ for 1 h. After the carboxyl group activated, compound 4 (0.56 g, 0.79 mmol) was added to the solution and stirred for 12 h at room temperature. The solution was extracted by ethyl acetate after the reaction was completed. The solvent was removed under reduced pressure to obtain the crude product. The crude product was purified by column chromatography on silica gel using DCM and methanol (10:1, v/v) as eluent, to obtain a blue solid powder. (0.48 g, 67.04 %).

^{1}H NMR (500 MHz, Chloroform-d) δ 7.63 (d, J = 40.0 Hz, 2H), 7.33 (d, J = 20.0 Hz, 4H), 7.07 (s, 2H), 6.92 (t, J = 7.5 Hz, 2H), 5.79 (d, J = 30.0 Hz, 2H), 3.85 (s, 4H), 3.74 (m, 4H), 3.12 (m, 4H), 2.94 (t, J = 17.5 Hz, 2H), 2.86 (t, J = 17.5 Hz, 2H), 2.76 (t, J = 5.0 Hz, 2H), 2.59 (t, J = 5.0 Hz, 2H), 2.44 (s, 4H), 1.77 (m, 6H), 1.63 (s, 12H), 1.22 (s, 52H), 0.89 (t, J = 5.0 Hz, 3H). HRMS calculated for $C_{72}H_{113}N_4O_3S_2^+$ 1145.82486, found 1145.82424.

Synthesis of compound 6.

Compound 5 (0.33 g, 1.58 mmol) and HATU (0.32 g, 3.79 mmol) were dissolved in 10 mL of DMSO, add DIPEA (0.18 g, 6.32 mmol) and stirred at 25 ℃ for 1 h. After the carboxyl group activated, chitosan oligosaccharide (0.16 g, 3.03 mmol) soluble in a small amount of water was added to the solution and stirred for 12 h at 25 ℃. After the reaction was completed, the reactant mixture was poured into excess acetone and centrifuged at 4000 rpm for 10 min to remove unreacted hexadecanol. The above precipitate step was repeated three times. Next, the precipitate was dispersed with deionized distilled water (DDW) and dialyzed with a dialysis membrane (MWCO= 3.5 kDa) against DDW for 48 h. Finally, the dialyzed products (CSO-SS-Cy7-Hex) were lyophilized.

Synthesis of SPION-Loaded CSO-SS-Cy7-Hex/SPION and sorafenib-Loaded CSO-SS-Cy7-Hex/Srfn.

SPION have been prepared as previously.[1] The SPION-loaded self-assemblies were prepared using the O/W emulsion solvent evaporation method. Typically, 1 mL of SPION (5 mg) in $CHCl_3$ was added to 10 mL of 10 mg of the amphiphilic copolymers CSO-SS-Cy7-Hex aqueous solution. The two-phase suspension was emulsified using a probe-type ultra sonicator (VCX-500; SONICS and MATERIALS, INC., USA) at 150 W for 10 min. The solution was vigorously stirred at 25 ℃ for one night to evaporate the organic solvent. Eventually, the CSO-SS-Hex/SPION assembly solution was obtained. Then the micellar solution was filtered through a 0.45 μm membrane to remove any large aggregates. The CSO-SS-Cy7-Hex/SPION was obtained by freeze-drying. The preparation of Hex-SS-Cy7-Hex/Srfn assembly was similar to CSO-SS-Hex/SPION, but the sorafenib were substituted for SPION.

Synthesis of CSO-SS-Cy7-Hex/SPION/Srfn and CSO-SS-Cy7-Hex/SPION/NR self-assembly.

The procedure for preparing the sorafenib and SPION loaded magnetic complex self-assemblies was divided into several steps. First, the loading of SPION into the CSO-SS-Cy7-Hex self-assemblies followed the O/W emulsion solvent evaporation method as described. Next, sorafenib was transferred into the magnetic self-assemblies using a dialysis technique. Briefly, 5 mg sorafenib was dissolved in 1 mL DMSO, and added to the solution of magnetic self-assemblies drop-by-drop under vigorous stirring for 15 min at room temperature. The mixture was ultra-sonicated for 15 min in an ice-bath using aprobe-type ultrasonicator at 150 W followed by dialysis against DDW for 12 h using a dialysis

bag (MWCO = 3.5 kDa). The crude product solution of CSO-SS-Cy7-Hex/SPION/Srfn was obtained. Afterwards, the CSO-SS-Cy7-Hex/SPION/Srfn micellar solution were removed from the dialysis bag and filtered through a 0.45 μm membrane to remove large aggregates and unloaded sorafenib. The drug loading (DL) and entrapment efficiency (EE) of sorafenib in the assembly was detected using an ultraviolet spectrophotometer. The DL and EE were calculated as the method previously. The content of SPION loaded in self-assemblies was determined using Perls stain soluble kit.

To visualize the intracellular release of cargo from the magnetic complex self-assemblies, the fluorescence probe Nile red (NR) was loaded into self-assemblies in accordance with the protocol for the preparation of the sorafenib-loaded self-assemblies (0.3%, W/W), separate from the change of sorafenib to Nile red and obtained CSO-SS-Cy7-Hex/SPION/NR.

In Vitro Drug Release Behavior

The *in vitro* drug release profiles were studied by dialyzing 2 mL of the magnetic complex assembly suspension (concentration of assembly was 1.0 mg/mL, contain 1% tween 80) in a dialysis bag (MWCO = 3.5 kDa) with 150 mL of PBS buffer (0.01 M, contain 1% tween 80) at pH 7.4 ± 10 mM GSH at 37 ℃ under gentle shaking (200 rpm). At selected time intervals: 0, 1, 2, 4, 8, 12, 24 and 48 h, 20 μL (the volume can be negligible, because it is far less than 2 mL) of in bag medium was withdrawn and replaced with an equal volume of fresh in bag medium, because the concentration of sorafenib and SPION out of bag medium were too low to be detected.[2] The collected samples were extracted by 100 μL methanol and analyzed by UV−vis spectrometer (266 nm) to determine the amount of released sorafenib. sorafenib release from stock solution was used as a control. The SPION concentrate was detected using Perls stain soluble kit.

Hysteresis Loop Measurement

The magnetization data for SPION and CSO-SS-Cy7-Hex/SPION/Srfn were determined using a vibrating sample magnetometer (VSM, VSM-175, China) at room temperature. The applied magnetic field was varied from 1.5 T to -1.5 T to generate hysteresis loops. The magnetic responsiveness of the CSO-SS-Cy7-Hex/SPION/Srfn self-assemblies in aqueous solution was tested by simply placing a magnet near the glass vial. A cylindrical sintered N38 NiCuNi-Fe magnet (d= 15 mm, h= 6 mm; the field strength is approximately 0.2 T) was used.

X-ray Diffraction (XRD) assay

The XRD pattern was recorded with a Rigaku D/max 2500 X-ray powder diffractometer using Cu Ka (1.54 Å) radiation (40 kV, 40 mA). The sample was scanned from 20° to 90° at a speed of 5° min^{-1}.

***In Vitro* Cell Uptake Assay.**

The 4T1 and MDA-MB-231 cells were seeded in 20 mm confocal dish and incubated for 24 h. Then CSO-SS-Cy7-Hex/SPION/Srfn complex self-assemblies (100 μg/mL) was added into each well and incubated for another 3 h, 6 h, 9 h, 12 h and 24 h respectively. After that, the cells were washed by PBS three times and 4% paraformaldehyde fixation 15 min before visualized using CLSM 700.

Flow cytometer was used to investigate the uptake of cancer cells. 4T1 cells were incubated with 100 μg/mL CSO-SS-Cy7-Hex/SPION/Srfn complex self-assemblies for 3 h, 6 h, 9 h, 12 h and 24 h, and collected to detected by flow cytometer.

GSH-responsive Assay

MDA-MB-231 cells were seeded in DMEM supplemented with 10% fetal bovine serum in confocal dish for 24 h. When the density reached 70−80%, the cells were pre-incubated by NEM (1mM) or GSH (10 mM) for 0.5 h. After washing with PBS, the cells were further incubated with CSO-SS-Cy7-Hex/SPION/Srfn (100 μg/mL) for 3 h and DAPI for 15 min at 37 ℃. Washed with PBS three times, and 4% paraformaldehyde fixation for 15 min. The cells were imaged using CLSM 700.

Magnetic Target of the HA/CSO-SS-Hex/SPION/NR complex self-assemblies

MDA-MB-231 cells were seeded in a 60-mm petri dish and maintained in 3 mL of DMEM medium supplemented with 10% FBS. After incubation for 24 h in a humidified incubator (37℃, 5% CO_2, 100 μg/mL of CSO-SS-Cy7-Hex/SPION/NR assembly was added. To evaluate the magnetic targeting properties of the self-assemblies, a magnet (Dimension: d = 15 mm, h = 6 mm; field strength approximately 0.2 T) was placed against the outer bottom wall of the petri dish, and the cells were incubated for an additional 3 h. The petri dish was washed three times with PBS, and Nile red fluorescence was analyzed using an OLYMPUS IX53 reflected fluorescence microscope. Using Perls stain soluble kit to do the Prussian blue staining experiment for 30 min at 37℃ to fix the cells and again washed once with PBS three times. And the cells were analyzed by a OLYMPUS IX53 reflected fluorescence microscope.

***In Vitro* ROS Study.**

The *in vitro* ROS generation was measured by the fluorescence change resulting from the oxidation of DCFH-DA (ROS-sensitive probe, 2′,7′-dichlorofluorescin Diacetate) by ROS. The 4T1 cells were

seeded into a 6-well plate at 1.0×105 cells per well and allowed to grown overnight at 37℃ in a humidified 5% CO_2 atmosphere. After that the cells were treated with different groups (DMEM, CSO-SS-Cy7-Hex, CSO-SS-Cy7-Hex/Srfn, CSO-SS-Cy7-Hex/SPION, double CSO-SS-Cy7-Hex/ SPION/Srfn) for 2 h, then light illuminate one of CSO-SS-Cy7-Hex/SPION/Srfn group with 808 nm laser (2.60 W/cm^{-2}) for 2 min and continue incubation the whole groups for another 1 h. Then the DCFH-DA (10 μM) in the serum-free DMEM was added and co-incubation for 30 min, after the cells were washed with PBS three times, the nuclei were stained with DAPI before CLSM700 observation. Flow cytometer was used to investigate the level of ROS in cancer cells. 4T1 cells were incubated with different complex self-assemblies for 24 h, incubated with DCFH-DA (10 μM) for 30 min, and collected to detected by flow cytometer.

Colocalization into Mitochondria.

MDA-MB-231 cells and 4T1 cells were seeded in a confocal dish for a day time. Then, CSO-SS-Cy7-Hex/SPION/Srfn (100 μg/mL) was delivered into the cells in DMEM culture medium. After incubation for 12 h, Cells were then washed with PBS for three times to remove the nanoparticles, which were not uptake into the cells. Then, 2 mL fresh DMEM culture medium was added and the cells were stained by Mito-Tracker Green (25 nM) at 37℃ for 15 min. The cells were then washed by PBS twice and immediately observed using confocal laser scanning microscopy (CLSM) and confocal images of cells fluorescence were quantified using the Image-Pro Plus Imaging software.

Luciferase labled 4T1 tumor-bearing mouse models were tumor injected by the mix solution of CSO-SS-Cy7-Hex/SPION/Srfn and mitochondrial probe, and extracted tumor. Slice up the frozen

section of tumor tissue, observed by CLSM700.

Western Blot Analysis.

4T1 cells were seeded on six 60-mm petri dishes and cultured at 5% CO_2, 37 ℃ overnight. (DMEM, CSO-SS-Cy7-Hex, CSO-SS-Cy7-Hex/Srfn, CSO-SS-Cy7-Hex/SPION, Light/No Light CSO-SS-Cy7-Hex/SPION/Srfn) were added and cultured at 100 μg/mL^{-1} for 24 h. The cell lysates were collected and analyzed by using a SDS-PAGE Gel Quick Preparation Kit. The protein bands were visualized using an enhanced Ultrasensitive ECL Chemiluminescence instrument.

***In Vitro* Cell Viability Assay**

Firstly, the breast cancer cells 4T1 cells, MCF-7 cells and MDA-MB-231 cells were seeded in 96-well plates and cultured at 5% CO_2, 37 ℃ for 24 h. When cell density reaches 70-80%, different groups (DMEM, CSO-SS-Cy7-Hex, CSO-SS-Cy7-Hex/Srfn, CSO-SS-Cy7-Hex/SPION, double CSO-SS-Cy7-Hex/SPION/Srfn) for 12 h, then illuminate one of CSO-SS-Cy7-Hex/SPION/Srfn group with 808 nm laser (2.60 W/cm^{-2}) for 2 min and continue incubation the whole groups for another 12 h. After that, the *in vitro* cytotoxicity was quantitatively analyzed by standard MTT assay.

Secondly, After the cells were seed in 96-well plates and grow for 24 h, the ferroptosis inhibitors, including DFO (200 μM) and baicalein (10 μM) were added to the cells for 1 h and then incubated with different groups for 12 h and illuminate for 2min and continue incubate for another 12 h. Finally, the cells were washed with PBS for 3 times. MTT reagent was added and incubated at 37 ℃ for another 4 h. A microplate reader was used to measure the absorbance.

Hydrogen peroxide assay

Hydrogen peroxide kit was used to detected the content of hydrogen peroxide in breast cancer cells incubated by CSO-SS-Cy7-Hex/SPION/Srfn self-assembly (100μg/mL) with illumination and DMEM for 24 h.

Hydroxyl radical assay

4T1 cells were incubated with CSO-SS-Cy7-Hex/SPION/Srfn self-assembly (100μg/mL) with illumination and DMEM for 24 h, lysed cells, then hydroxyl radical kit was used to detected the content of hydroxyl radical in it.

Intracellular GSH Content.

Breast cancer cells (4T1 cells, MDA-MB-231 cells and MCF-7 cells) were seeded on a cell-culture 6-plates dish and cultured at 5% CO_2, 37 ℃ overnight. Different samples (DMEM, CSO-SS-Cy7-Hex, CSO-SS-Cy7-Hex/Srfn, CSO-SS-Cy7-Hex/SPION, Light/No Light CSO-SS-Cy7-Hex/SPION/Srfn) were added and cultured for 24 h at 100 μg/mL^{-1}. The glutathione content was measured using a glutathione Assay kit and the absorbance were measured by a microplate reader.

Lipid peroxidation assay

Breast cancer cells were incubated in complex self-assemblies, and the relative malondialdehyde (MDA) concentration in cell lysates was assessed using a Lipid Peroxidation (MDA) Assay Kit according the instructions.

Iron assay

The relative iron concentration in cell lysates was assessed using an Perls stain soluble kit according to the manufacturer's instructions.

Carbonylation protein assay

4T1 cells were incubated with different self-assembles (DMEM, CSO-SS-Cy7-Hex, CSO-SS-Cy7-Hex/Srfn, CSO-SS-Cy7-Hex/SPION, Light/No Light CSO-SS-Cy7-Hex/SPION/Srfn) for 24 h, then carbonylation protein kit was used to detected the content of carbonylation protein in cells.

Apoptotic experiment

4T1 cells were incubated with the CSO-SS-Cy7-Hex/SPION/Srfn (50 μg/mL) complex self-assembly for 12 h, flow cytometry was used to evaluate the apoptotic of cells.

Photothermal assay

4T1 cells were incubated with the CSO-SS-Cy7-Hex/SPION/Srfn (100 μg/mL) complex self-assembly for 24 h, collected and laser irradiated for 5 min, NIR instrument was used to evaluate the temperature variation of cells.

Complex Self-assembly Suppresses TGF-β1-induced EMT in Triple negative breast cancer cells

Treatment with complex counteracts TGF-β1 induced EMT in breast cancer cells. 4T1 cells,

MDA-MB-231 cells and MCF-7 cells were seed in 6-plates and treated with TGF-β1 (5 ng/mL) for 48 h to make the cells to mesenchymal cells, then incubate cells with different groups (DMEM, CSO-SS-Cy7-Hex, CSO-SS-Cy7-Hex/Srfn, CSO-SS-Cy7-Hex/SPION, double CSO-SS-Cy7-Hex/SPION/Srfn) for 12 h, after illuminate (2.60 W/cm^{-2}), continue incubation the whole groups for another 12 h. Finally, the cells were visualized using an OLYMPUS IX53 reflected fluorescence microscope.

Breast cancer cells were seed in 96-plates/6-plates and incubated with TGF-β1 (5 ng/mL) for 48 h and treated with Paclitaxel, Adriamycin, Gemcitabine, Camptothecin and the complex self-assemblies CSO-SS-Cy7-Hex/SPION/Srfn (100μg/mL) 12 h and illuminate (2.60 W/cm^{-2}) for 2min and incubated for another 48 h. The cytotoxicity was quantitatively analyzed by standard MTT assay or assessed using a Lipid Peroxidation (MDA) Assay Kit.

Anti-Cell-Migration Assay

To assess cell motility, 4T1 cells, MCF-7 cells and MDA-MB-231 cells (5×10^5 cells per well) were seeded in 6-well plates and cultured as confluent monolayers. The monolayers were scraped with a sterile yellow micropipette tip (0 h) to create a denuded zone with constant width and washed twice to remove cellular debris. The cells were exposed to (DMEM, CSO-SS-Cy7-Hex, CSO-SS-Cy7-Hex/Srfn, CSO-SS-Cy7-Hex/SPION, Light/No Light CSO-SS-Cy7-Hex/SPION/Srfn) for 24 h. At 0 and 24 h, the scratched monolayers were photographed using a OLYMPUS IX53 reflected fluorescence microscope, and the scratch width was determined.

***In Vivo* Tumor Magnetic Targeting Imaging Assay**

BALB/c female nude mice injected with MCF-7 cells (1×10^5) to make tumor model. When the tumors were grown to an average volume of 100 mm^3 (the tumor volume was calculated by the formula: $V = L \times W^2/2$, where L and W stand for the maximum and minimum diameter of the tumors) in diameter before the experiment. The CSO-SS-Cy7-Hex/SPION/Srfn self-assembly solution were injected with the concentration of sorafenib 6 mg/kg and stick/no stick a magnet (approximately 0.2 T) on the top of the tumor by tape. At 0.083, 1, 3, 6, 9 and 24 h post-injected, whole-body fluorescence images were acquired using a NIR fluorescence imaging system with a wavelength set at Ex =710 nm and Em =835 nm after the mice were anesthetized. After imaging the 24 h, the main organs (Tumor, Heart, liver, spleen, lung, kidney, stomach, intestine) were harvested and imaged immediately.

Pharmacokinetic Evaluation of sorafenib and CSO-SS-Cy7-Hex/SPION/Srfn

Pharmacokinetic studies were used Sprague Dawley male rats (200 g) bought by Qinglongshan animal breeding farm (Nanjing, China). The rats were given ad libitum access to food and water under standard laboratory conditions. The rats were divided into two groups (CSO-SS-Cy7-Hex/SPION/Srfn and sorafenib) and the drugs were administered to mice by caudal vein. The blood samples were obtained at a specified time (0.25, 0.5, 1, 2, 4, 8, 12, 24 and 48 h) in tubes, then the plasma samples (about 400 μL) were centrifuged at 8000 rpm for 10 min and stored at -80 ℃. Sorafenib was extracted with acetonitrile from 100 μL Plasma sample and analyzed by LC-MS. The pharmacokinetic parameters: $t_{1/2}$ (elimination half-life), CL (clearance), AUC (area under the concentration time curve) and MRT (mean residence time) were calculated by the software of DAS (Version 2.0).

***In Vivo* Tumor Inhibition.**

A tumor-bearing Female BALB/c mice model was established by subcutaneously injecting 4T1 tumor cells (1×10^5 cells suspended in 100 μL of PBS) into the flank of each mouse. When the tumors volume was grown to 150−200 mm^3, the mice were divided randomly into 6 groups (saline, CSO-SS-Cy7-Hex, CSO-SS-Cy7-Hex/Srfn, CSO-SS-Cy7-Hex/SPION, magnetic /No magnetic CSO-SS-Cy7-Hex/SPION/Srfn (6 mg/kg sorafenib). After injected the self-assemblies, one group was stick a magnet (approximately 0.2 T) on the top of the tumor by tape, then the whole groups were cultured for 12 h, and irradiated with a laser of 2.6 W/cm^{-2} for 5 min. After that, continue culture the mice for another 12 h, then the tumor volume and body weight of each mouse was recorded. Repeat the above cycle on the third day after the first administration. At the 11th day post-injection, all of the mice were sacrificed, and the tumor organs were harvested and preserved in a 4% paraformaldehyde solution.

***In Vivo* Biosafety Evaluation**

ICR female mouse (18-20 g) were randomly divided into three groups to receive IV injection of ddH_2O (control group), sorafenib solution (6 mg/kg) and CSO-SS-Cy7-Hex/SPION/Srfn (6 mg/kg) every three days, and two days after the third time, organs and blood were collected. The aspartate aminotransferase (AST) and alanine aminotransferase (ALT) level of serum using AST and ALT activity Assay Kit were evaluated to measure the liver function. The serum levels of urea nitrogen (BUN) and creatinine (CRE) using colorimetry according to the manufacturer's instruction was evaluated to measure the renal function. Major organs (heart, liver, spleen, lung and kidney) were

fixed in 4% paraformaldehyde for hematoxylin and eosin (H&E) staining, and the histology of five organs was assessed using Olympus microscope.

Statistical analysis

Unless otherwise indicated, the statistical analysis was processed using Graphpad Prism software (Version7.01) or Origin Pro software (Version 2015). The quantitative data were presented as mean ± standard deviation (SD).

Reference

[1] M. Sang; Z. Zhang; F. Liu; L. Hu; L. Li; L. Chen; F. Feng; W. Liu; W. Qu, *Journal of biomedical nanotechnology* 2018, *14*, 477.

[2] Y. Kang; L. Lu; J. Lan; Y. Ding; J. Yang; Y. Zhang; Y. Zhao; T. Zhang; H. Rjy, *Acta Biomaterialia* 2017.

Table S1. Properties of sorafenib or SPION-loaded complex assembles.

Concentration (μg/mL)	Sample	DL (%)	EE (%)
Sorafenib	CSO-SS-Cy7-Hex/Srfn	37.42 ± 0.23	79.25 ± 0.33
	CSO-SS-Cy7-Hex/SPION/Srfn	20.36 ± 2.02	82.22 ± 3.25
Iron	CSO-SS-Cy7-Hex/SPION	35.27 ± 2.43	96.56 ± 0.25
	CSO-SS-Cy7-Hex/SPION/Srfn	27.88 ± 1.92	96.23 ± 2.14

Table S2. Calculated 50% inhibiting concentration (IC50 of different formulations of different samples against 4T1, MCF-7 and MDA-MB-231 cell lines, n=3).

IC50 (Units)	Sample	4T1	MCF-7	MDA-MB-231
Sorafenib (μM)	Sorafenib	2.188 ± 0.52	0.872 ± 0.05	3.694 ± 0.10
	CSO-SS-Cy7-Hex/Srfn	0.046 ± 0.01***	0.019 ± 0.03***	0.082 ± 0.06***
	CSO-SS-Cy7-Hex/SPION/Srfn	0.035 ± 0.05***	0.024 ± 0.04***	0.067 ± 0.03***
	CSO-SS-Cy7-Hex/SPION/Srfn Light	0.022 ± 0.03***	0.009 ± 0.03***	0.012 ± 0.02***

Iron (μM)	CSO-SS-Cy7-Hex/SPION	0.535 ± 0.01	0.242 ± 0.01	0.265 ± 0.01
CSO-SS-Cy7 (μg/mL)	CSO-SS-Cy7-Hex	78.28 ± 1.31	73.28 ± 0.52	59.06 ± 0.1

Table S3. Pharmacokinetic parameters of CSO-SS-Cy7-Hex/SPION/Srfn assembles. (n=5), *p<0.05，**p<0.01.

Parameter	Units	CSO-SS-Cy7-Hex/SPION/Srfn	Sorafenib
$t_{1/2}$	h	8.13 ± 3.02**	1.06 ± 0.30
AUC $_{0\text{-}t}$	ng/ml*h	35290.47 ± 9996.12**	3627.59 ± 924.59
AUC $_{0\text{-}\infty}$	ng/ml*h	36340.98 ± 11194.28**	3627.59 ± 924.59
MRT	h	11.70 ± 4.37**	1.53 ± 0.43

Table S4. IC 50 of different antitumor drugs against 4T1 cell lines. (n=3)

Sample	Units	IC 50	IC 50 (EMT)
Paclitaxel	(μM)	2.74 ± 0.52	19.86 ± 0.28***
Adriamycin	(μM)	8.53 ± 0.23	32.27 ± 0.14**
Camptothecin	(μM)	6.10 ± 0.14	No
Gemcitabine	(μM)	9.76 ± 0.25	No
CSO-SS-Cy7-Hex/SPION/Srfn	(μM)	0.02 ± 0.04	0.003 ± 0.01

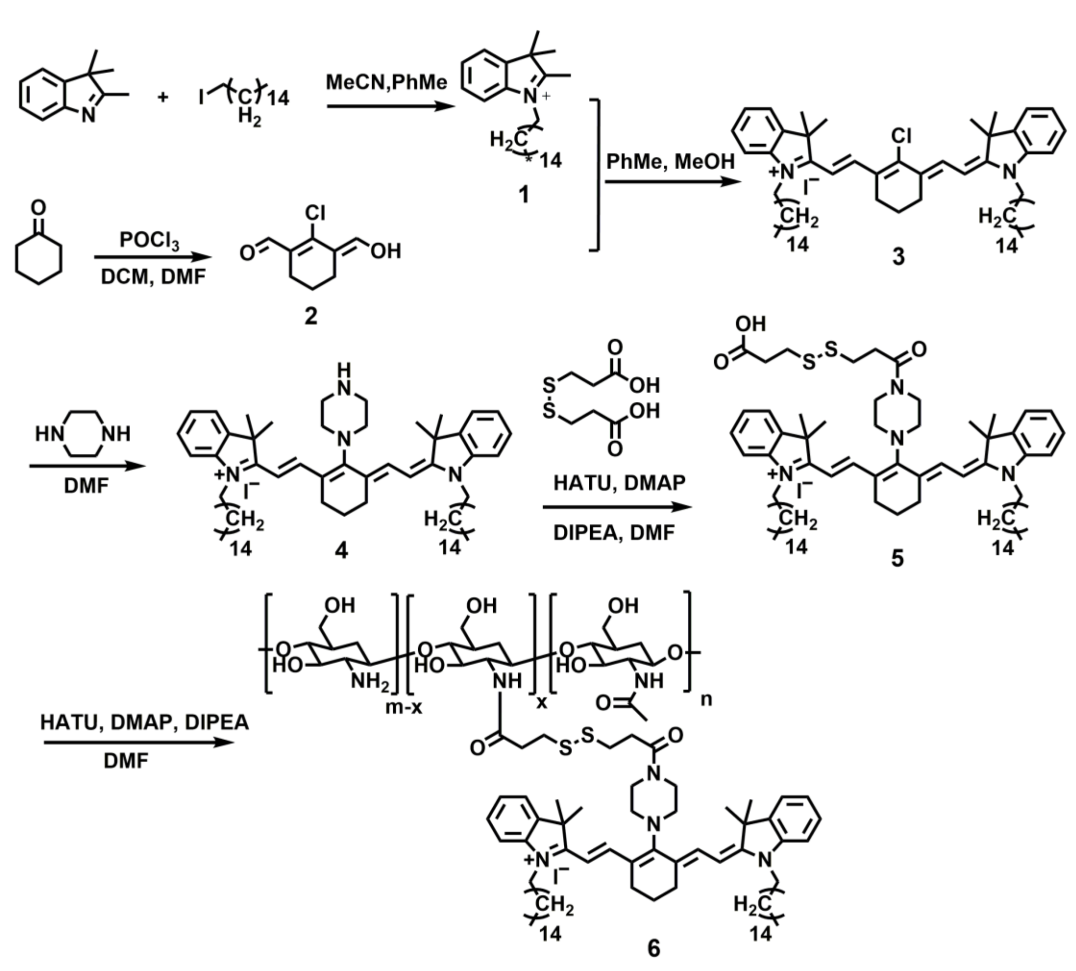

Figure S1. Synthesis route of CSO-SS-Cy7-Hex copolymer assembly.

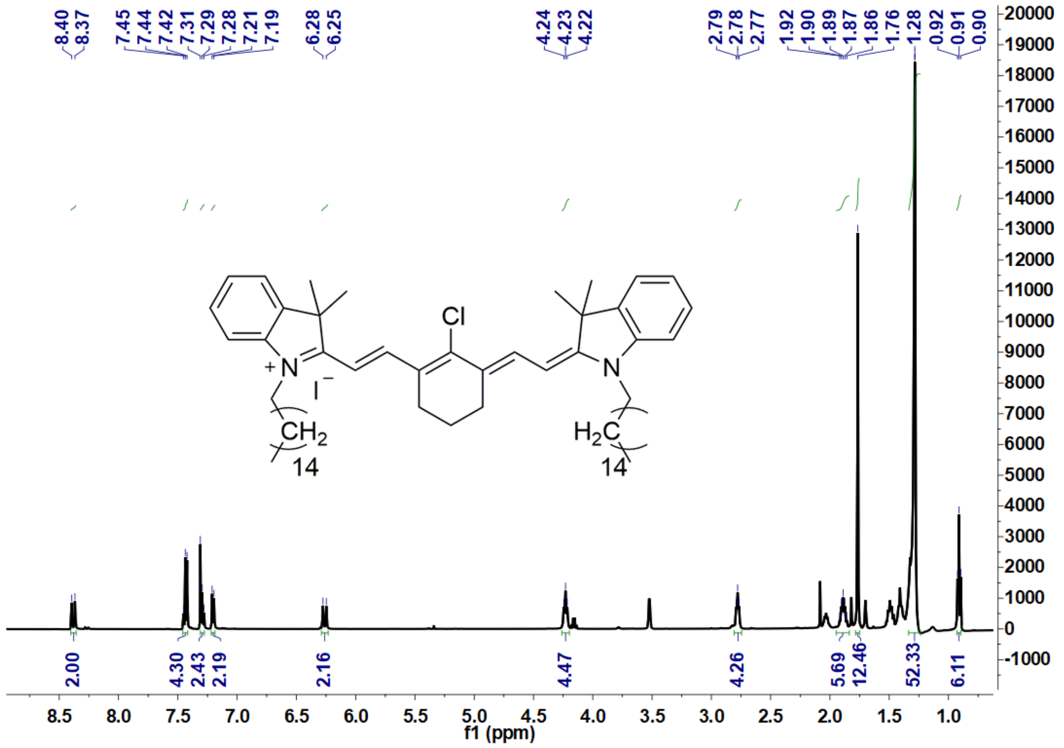

Figure S2. ^{1}H NMR (300 MHz) spectra of compound 3 in $CDCl_3$.

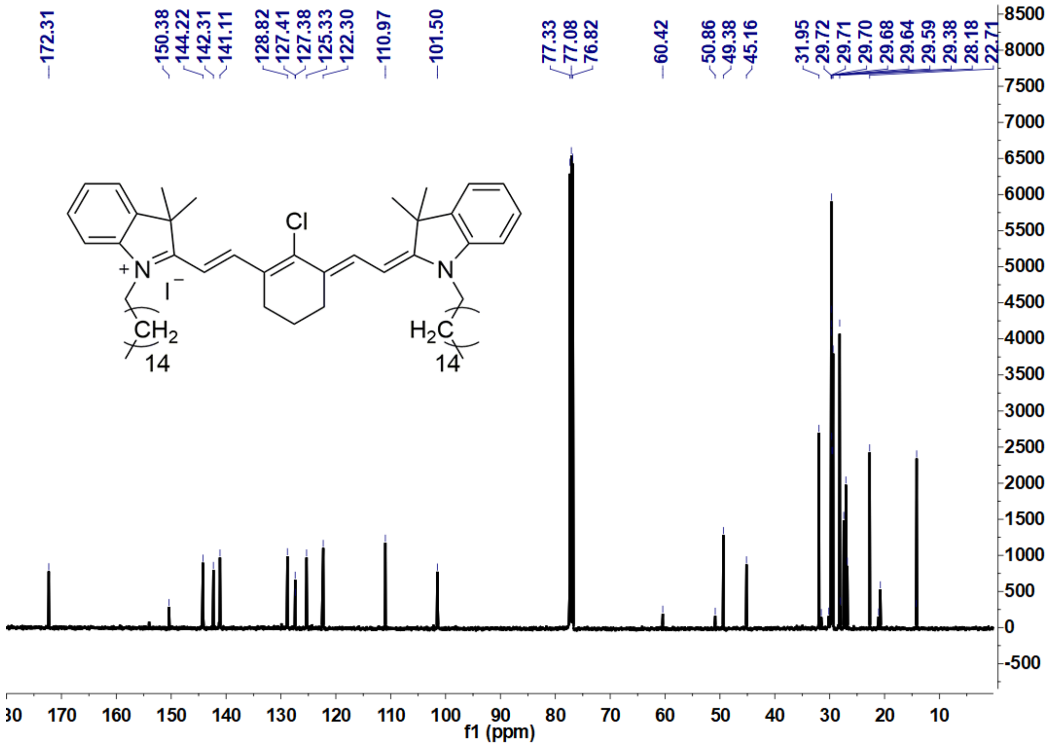

Figure S3. ^{13}C NMR (300 MHz) spectra of compound 3 in $CDCl_3$.

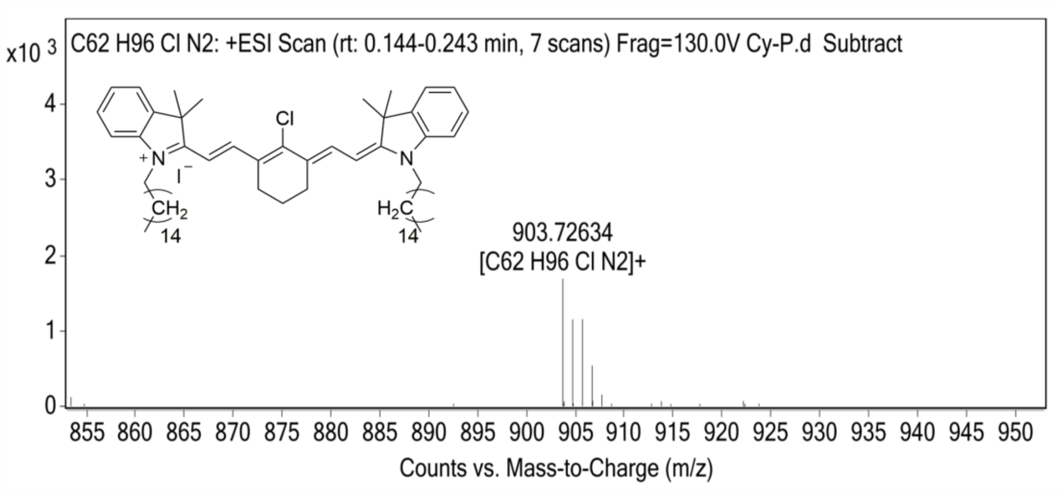

Figure S4. LC-MS (Positive Ion Mode) spectra of compound 3 in $CDCl_3$.

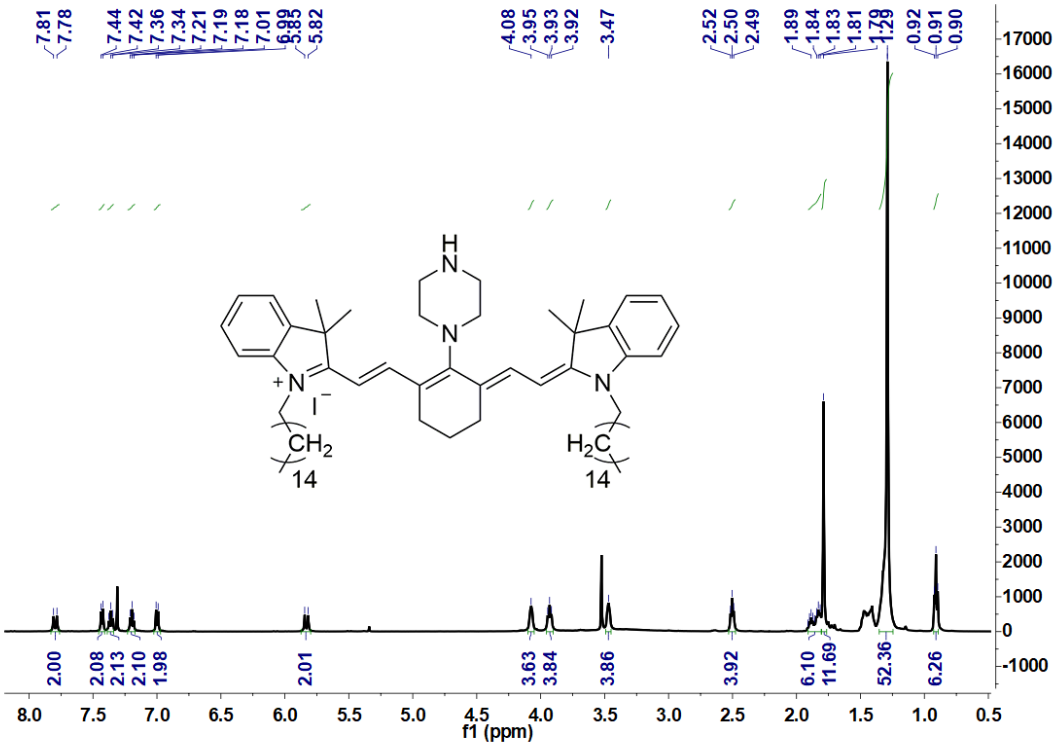

Figure S5. ^{1}H NMR (300 MHz) spectra of compound 4 in $CDCl_3$.

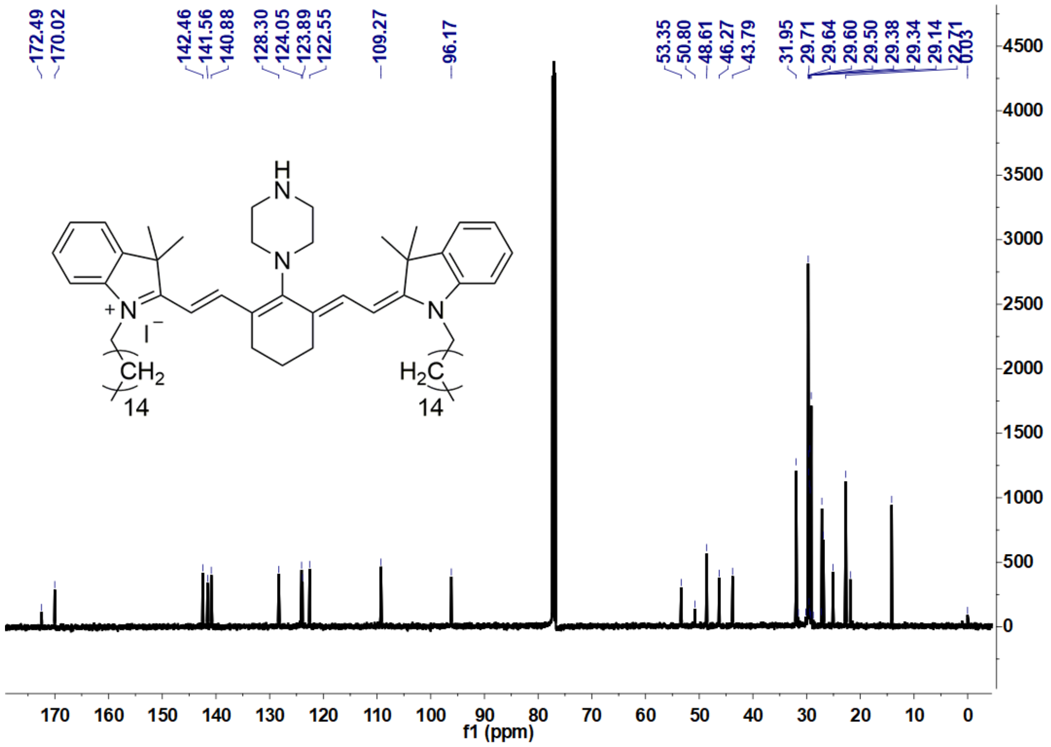

Figure S6. ^{13}C NMR (300 MHz) spectra of compound 4 in $CDCl_3$.

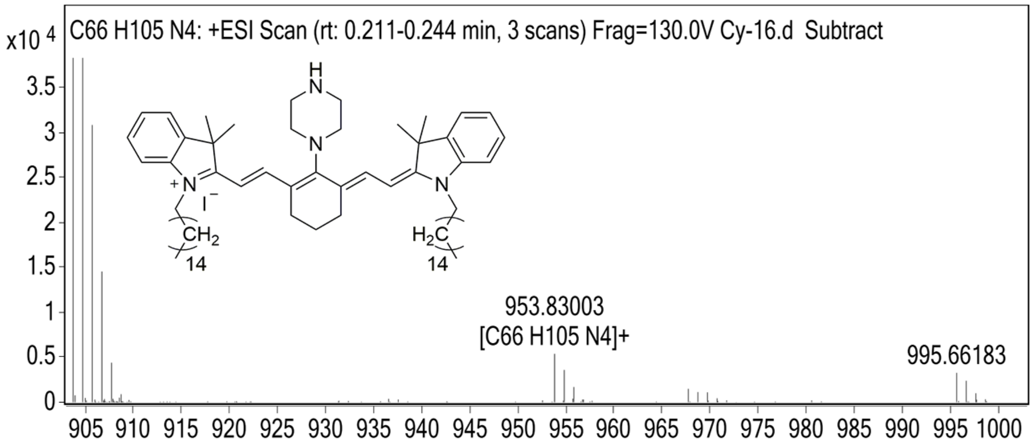

Figure S7. LC-MS (Positive Ion Mode) spectra of compound 4 in $CDCl_3$.

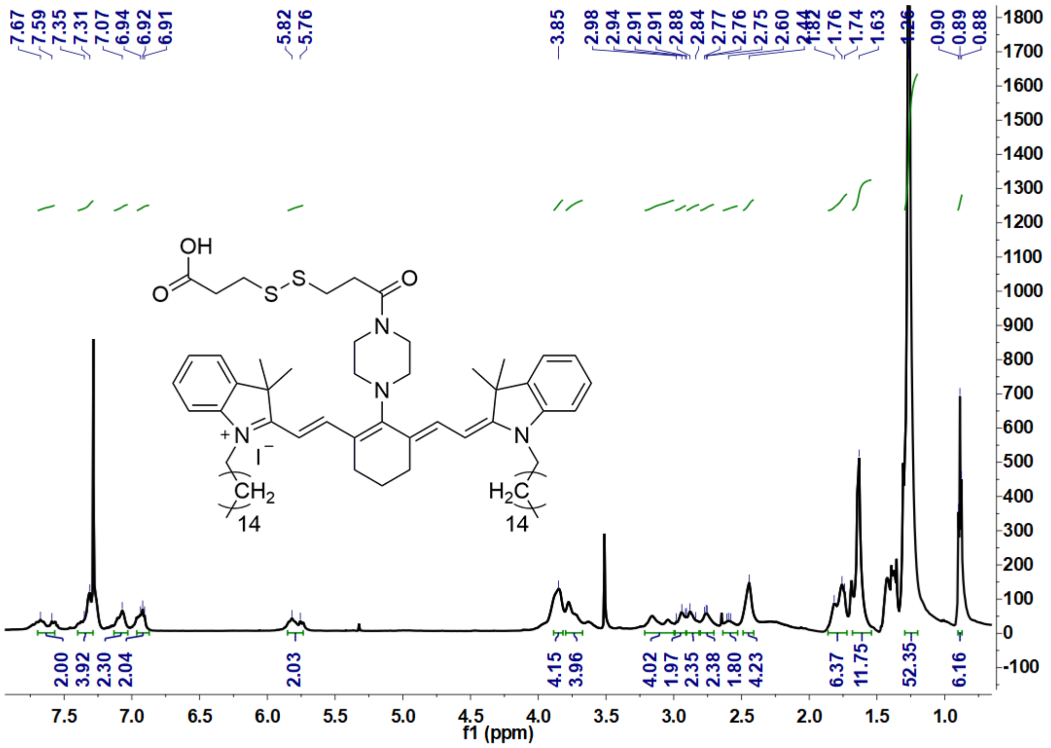

Figure S8. ^{1}H NMR (300 MHz) spectra of compound 5 in $CDCl_3$.

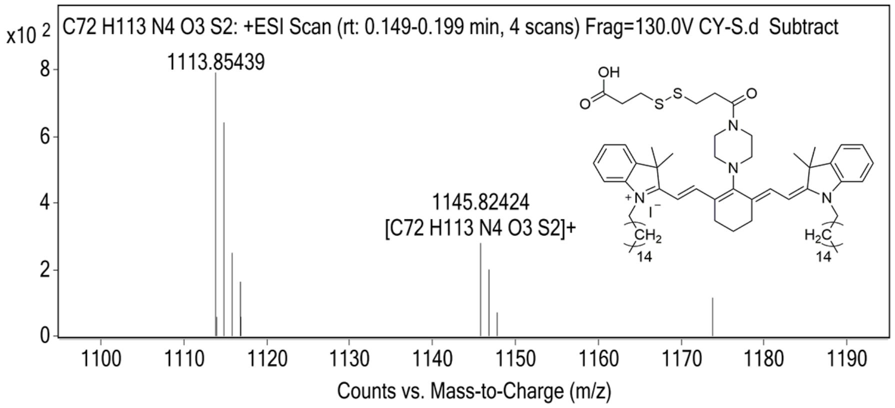

Figure S9. LC-MS (Positive Ion Mode) spectra of compound 5 in $CDCl_3$.

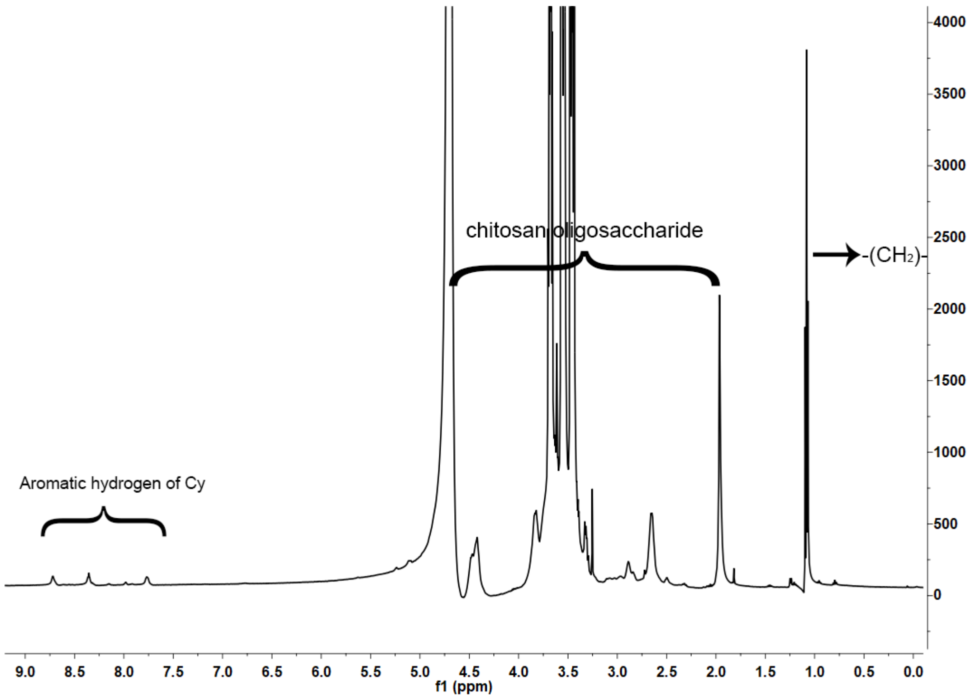

Figure S10. ^{1}H NMR (300 MHz) spectra of compound 6 in D_2O.

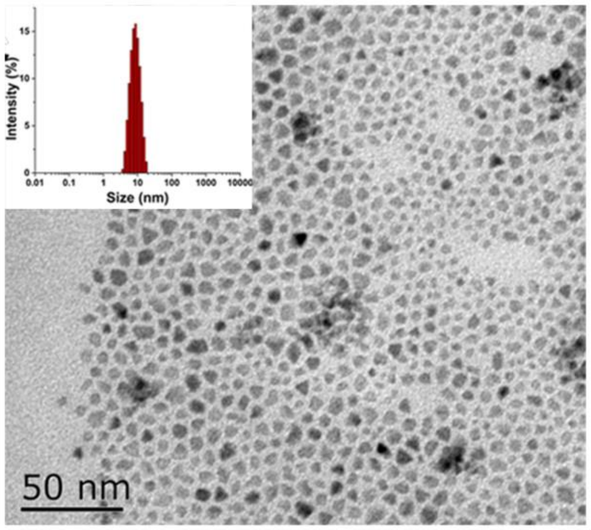

Figure S11. TEM and DLS images of SPION.

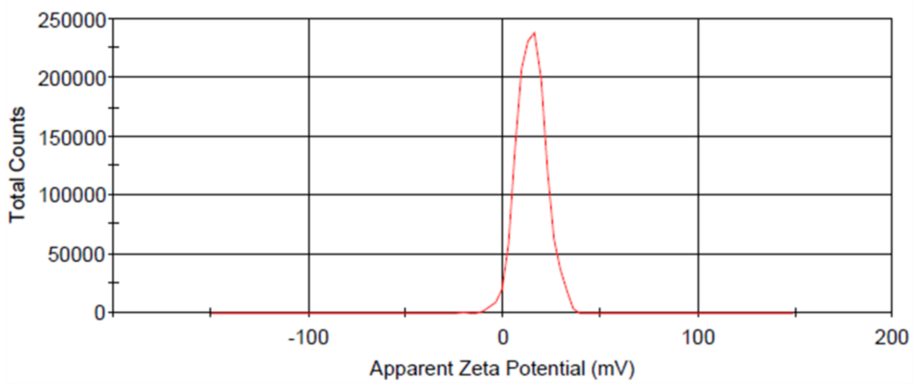

Figure S12. Zeta potential spectra of CSO-SS-Cy7-Hex/SPION/Srfn self-assemblies.

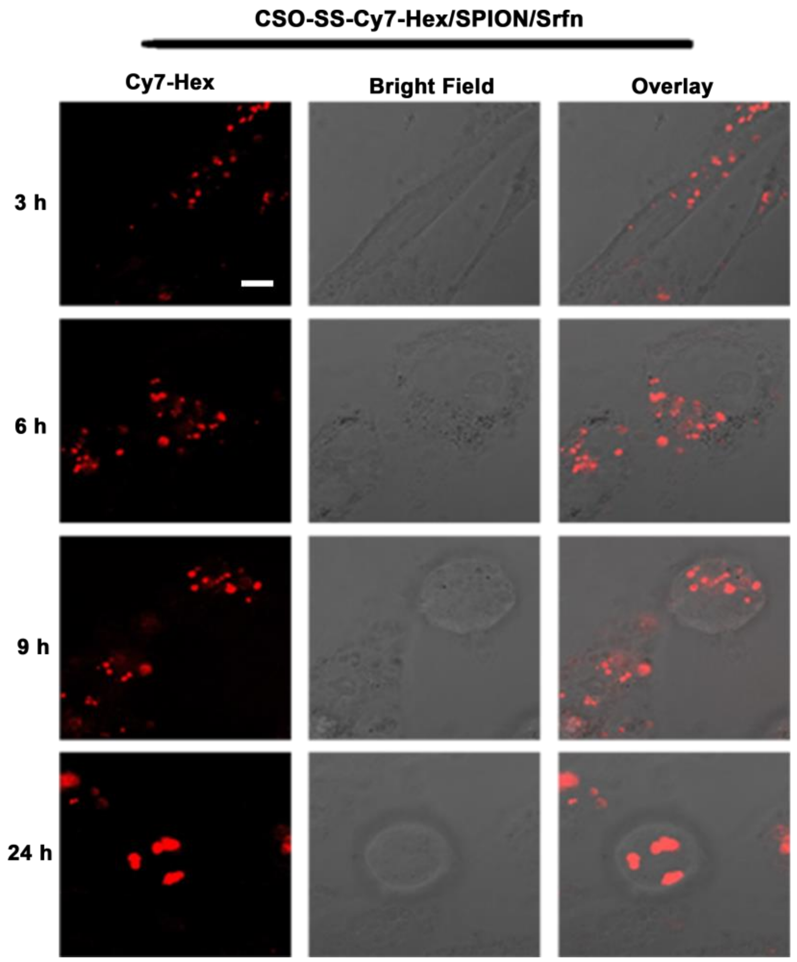

Figure S13. CLSM image of MDA-MB-231 cells incubated with CSO-SS-Cy7-Hex/SPION/Srfn at different times. (scale bar: 5 μm).

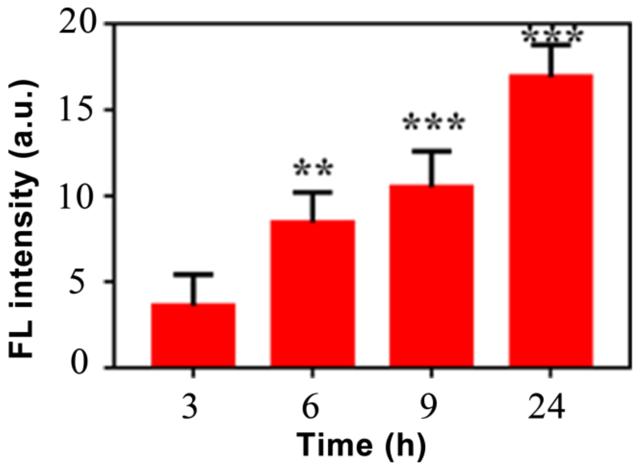

Figure S14. Fluorescence intensity of MDA-MB-231 cells incubated with CSO-SS-Cy7-Hex/SPION/Srfn at different times. (n = 3)

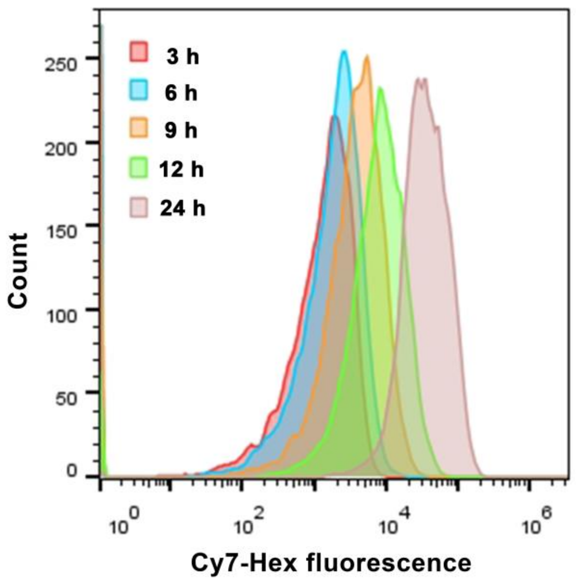

Figure S15. 4T1 cells incubated with CSO-SS-Cy7-Hex/SPION/Srfn complex self-assembly at different times and detected in flow cytometry.

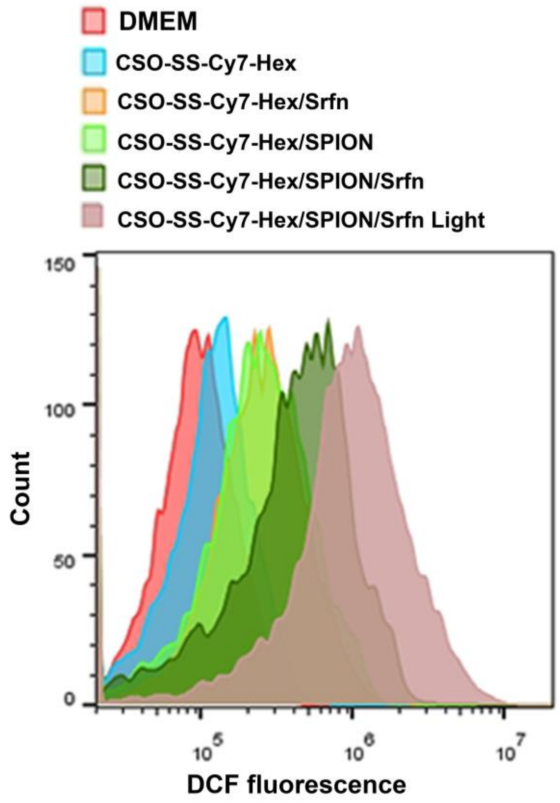

Figure S16. DCFH-DA assay of 4T1 cells treated with CSO-SS-Cy7-Hex/SPION/Srfn Light/No Light, CSO-SS-Cy7-Hex/Srfn, CSO-SS-Cy7-Hex/SPION, CSO-SS-Cy7-Hex, and DMEM to flow cytometry.

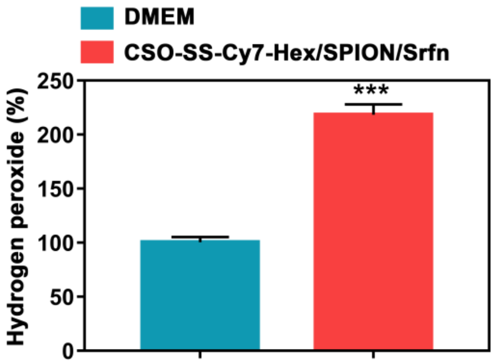

Figure S17. 4T1 cells incubated with CSO-SS-Cy7-Hex/SPION/Srfn with light self-assemblies for 24 hours and H_2O_2 levels were assayed (n=3).

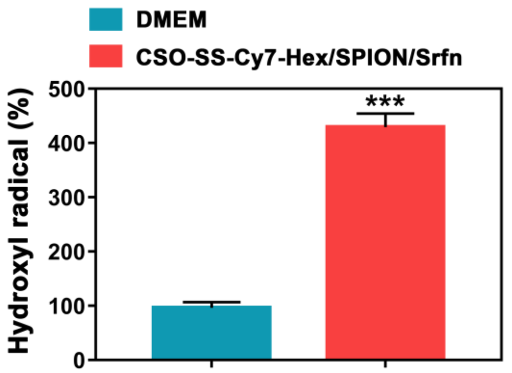

Figure S18. 4T1 cells incubated with CSO-SS-Cy7-Hex/SPION/Srfn with light self-assemblies for 24 hours and hydroxyl radical level were assayed (n=3).

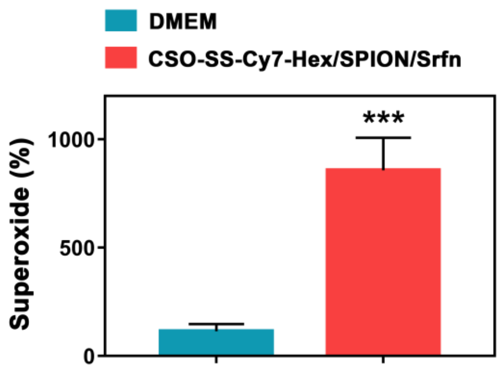

Figure S19. 4T1 cells incubated with CSO-SS-Cy7-Hex/SPION/Srfn with light self-assemblies for 24 hours and superoxide level were assayed (n=3).

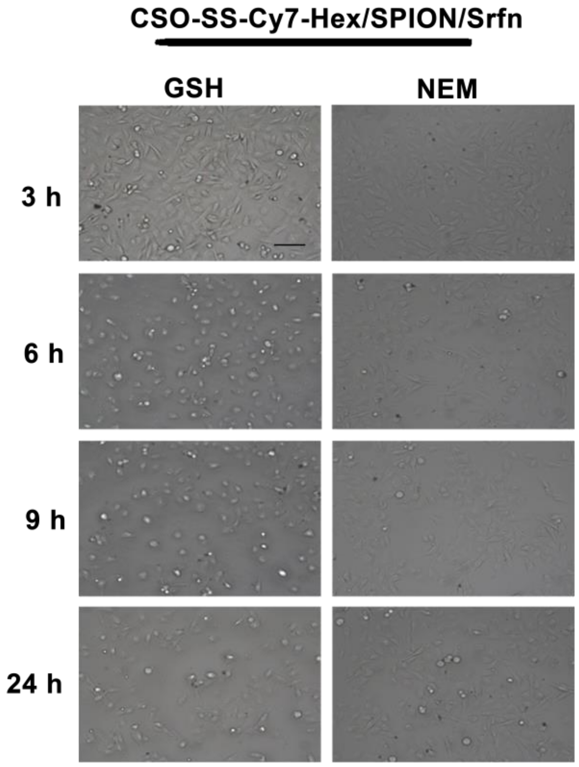

Figure S20. The GSH reduction response of CSO-SS-Cy7-Hex/SPION/Srfn self-assemblies. MDA-MB-231 cells were incubated with CSO-SS-Cy7-Hex/SPION/Srfn for different times after pretreated with NEM (1mM) and GSH (10mM). (scale bar: 50 μm).

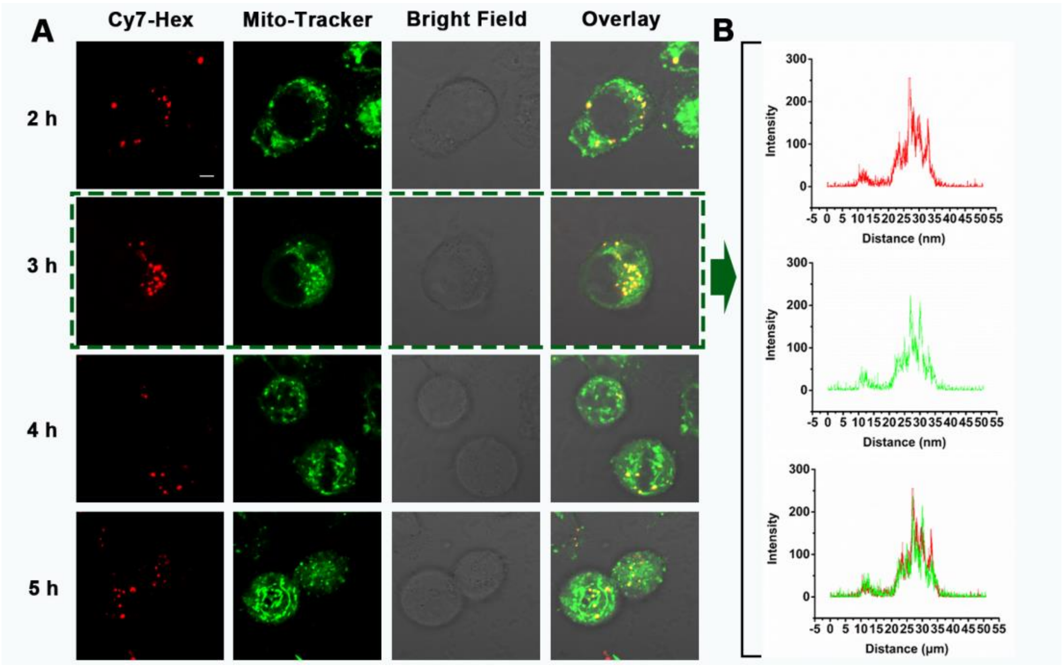

Figure S21. (A) CLSM images and linear profile of MDA-MB-231 cells were used to characterize the overlap degree of mitochondrion green fluorescence of Mito-Tracker and red fluorescence of NIR photosensitizer (scale bar: 5 μm). (B) MDA-MB-231 cells uptake of CSO-SS-Cy7-Hex/SPION/Srfn complex self-assemblies at different times to ferroptosis. The green fluorescence was Mitor-Tracker. (scale bar: 5 μm).

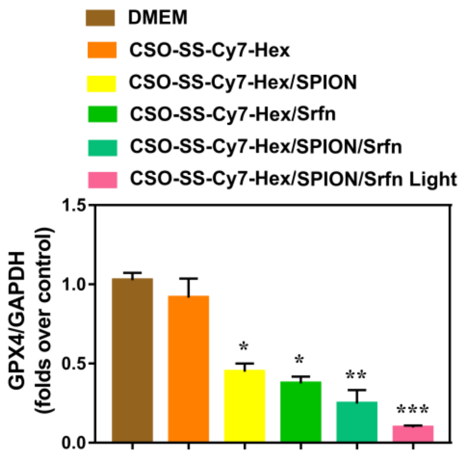

Figure S22. Relative western blot analysis of different self-assemblies. (n=3).

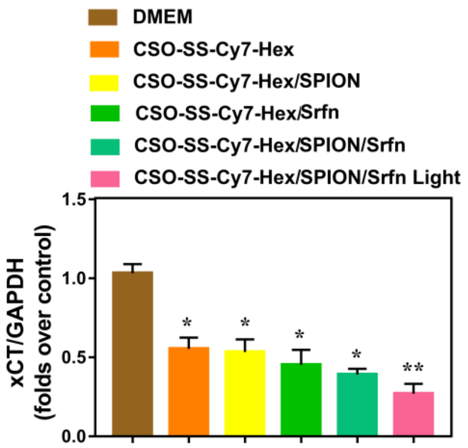

Figure S23. Relative western blot analysis of different self-assemblies. (n=3).

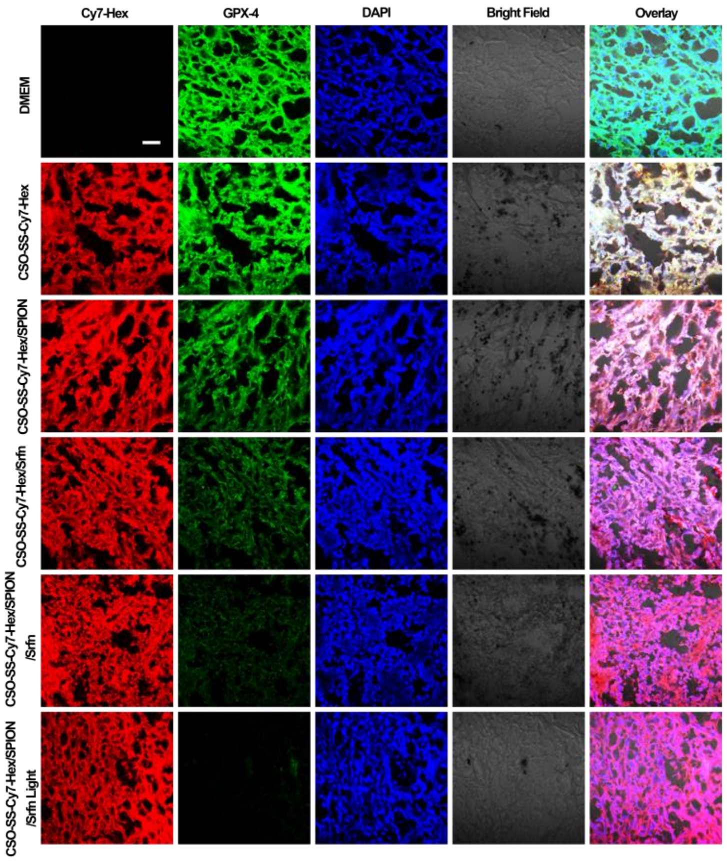

Figure S24. Immunofluorescence images of GPX-4 in tumor tissues after treated with different self-assemblies. (scale bar: 5 μm).

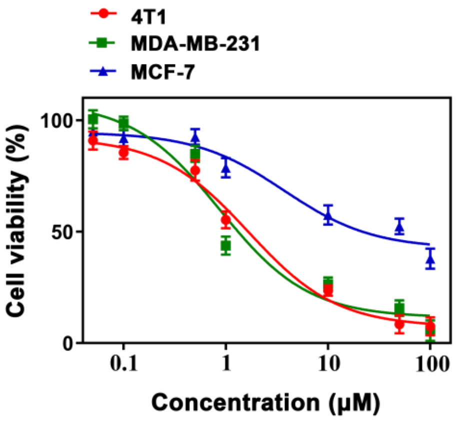

Figure S25. Cell viability of sorafenib treated 4T1 cells, MCF-7 cells, and MDA-MB-231 cells, respectively. (n=6).

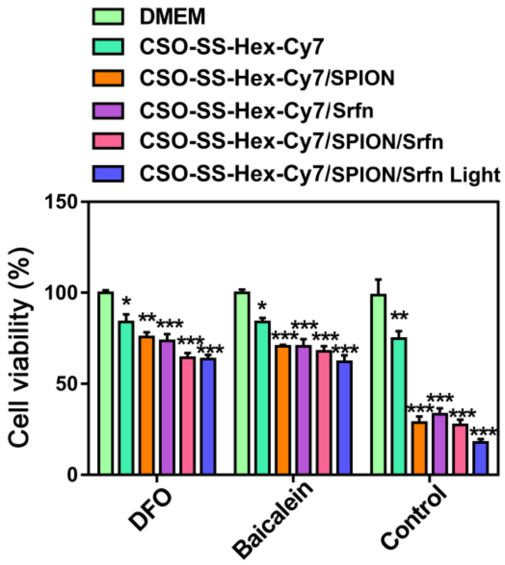

Figure S26. Relative cell viability of different self-assemblies treated MCF-7 cells after the addition of deferoxamine (DFO, 200 μM), Baicalein (10 μM) and DMEM, respectively.

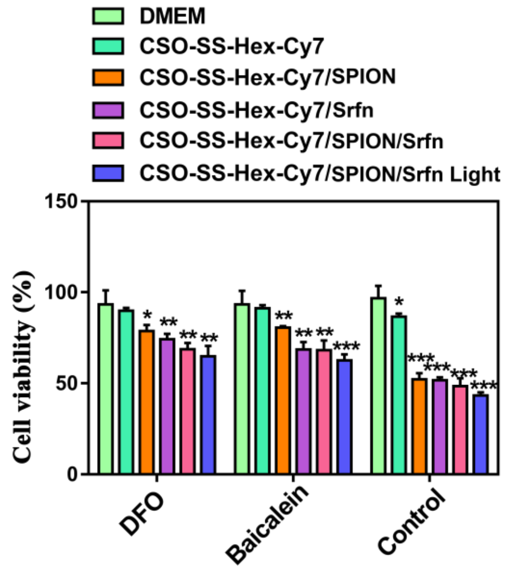

Figure S27. Relative cell viability of different self-assemblies treated MDA-MB-231 cells after the

addition of deferoxamine (DFO, 200 μM), Baicalein (10 μM) and DMEM, respectively.

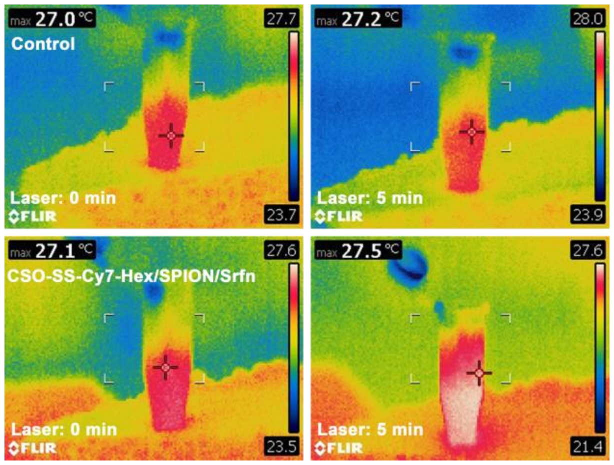

Figure S28. Temperature changed of the 4T1 cells incubated with CSO-SS-Cy7-Hex/SPION/Srfn self-assemblies (100 μg/mL) for 12 h and laser for 5 min.

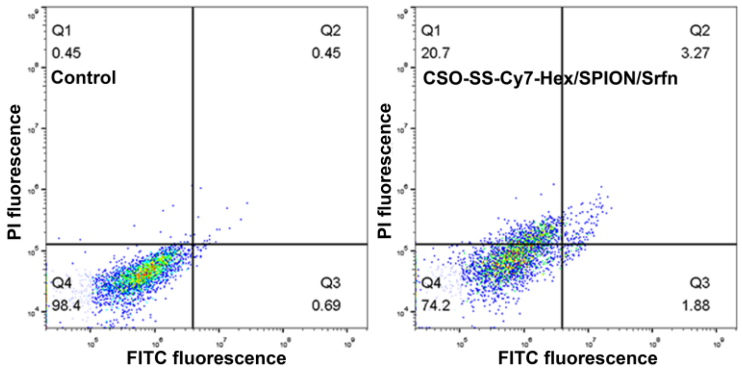

Figure S29. 4T1 cells incubated with CSO-SS-Cy7-Hex/SPION/Srfn self-assemblies for 12 hours and the cells apoptosis were assayed by flow cytometry.

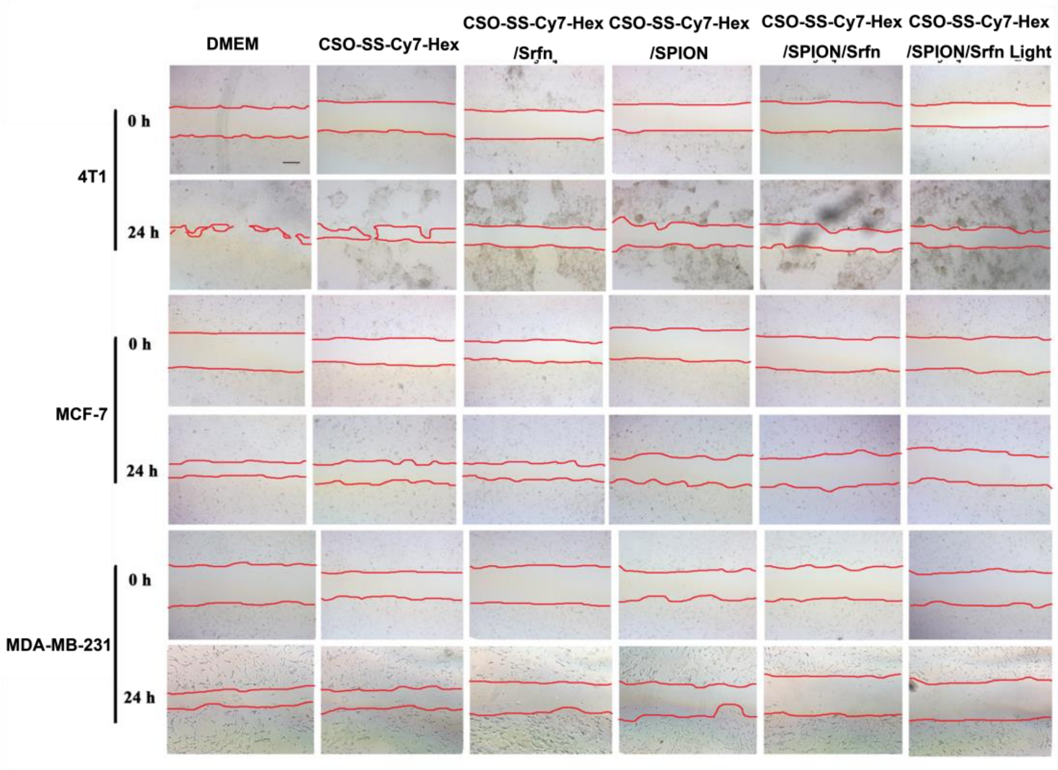

Figure S30. *In vitro* scratch assays to test cell migration. Scratch width of control, CSO-SS-Cy7-Hex, CSO-SS-Cy7-Hex/Srfn, CSO-SS-Cy7-Hex/SPION and Light/No Light CSO-SS-Cy7-Hex/SPION/Srfn treated 4T1, MCF-7, MDA-MB-231 cells (three cells lines were all treated with 5 ng/mL TGF-β1 for 48 h) at 0 h and 24 h. Wound edges are marked with red lines. Scale bars correspond to 200 μm in all images.

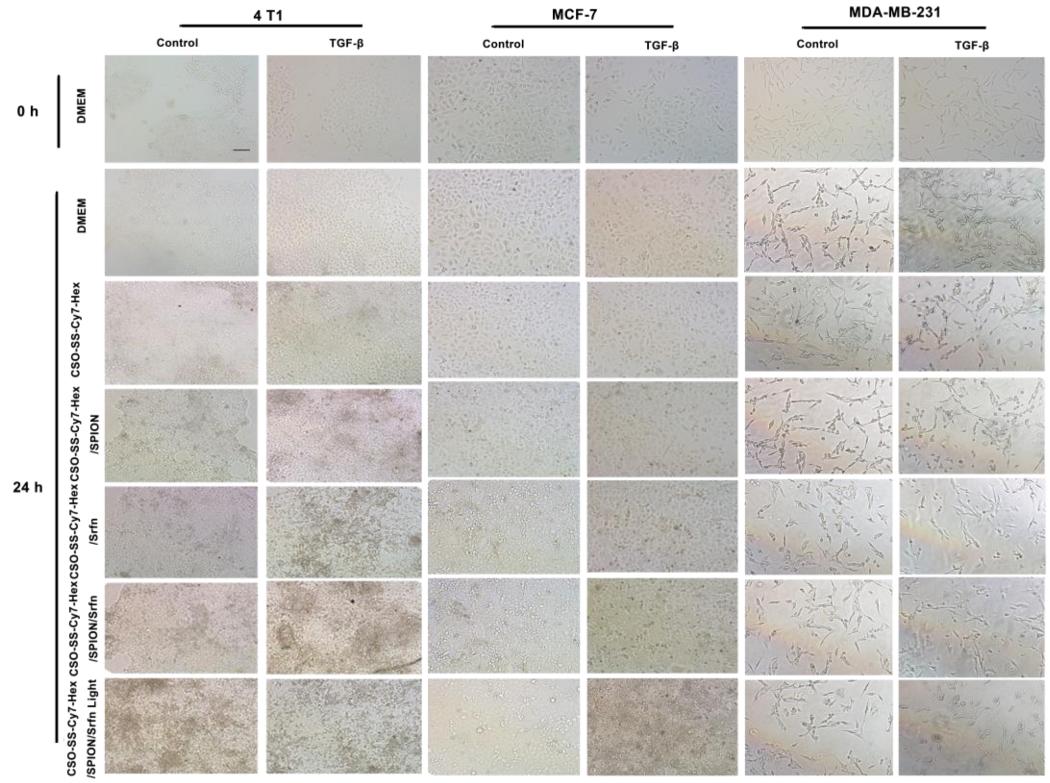

Figure S31. Treatment with control, CSO-SS-Cy7-Hex, CSO-SS-Cy7-Hex/Srfn, CSO-SS-Cy7-Hex/SPION and Light/No Light CSO-SS-Cy7-Hex/SPION/Srfn assembly counteracts TGF-β1-induced (5 ng/mL) EMT in 4T1, MCF-7 and MDA-MB-231 cells. The scale bar represents 200 μm for each section.

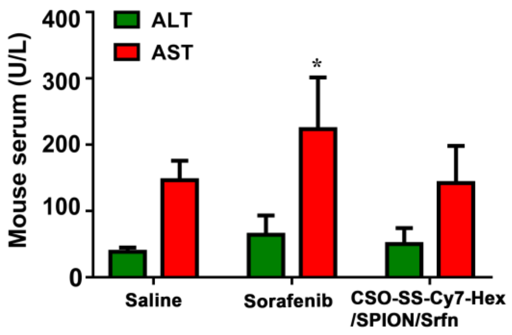

Figure S32. Biosafety of CSO-SS-Cy7-Hex/SPION/Srfn complex self-assemblies *in vivo*. Serum levels of ALT and AST (liver function) at 48 h after last treatment. (n=6).

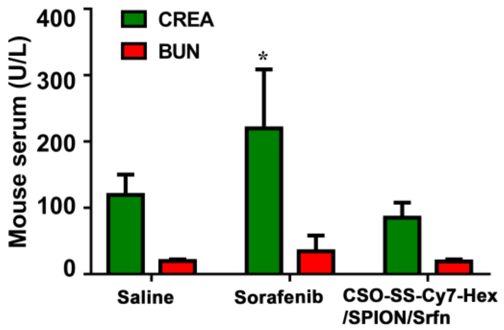

Figure S33. Biosafety of CSO-SS-Cy7-Hex/SPION/Srfn complex self-assemblies *in vivo*. Serum levels of BUN and CREA (renal function) at 48 h after last treatment. (n=6).

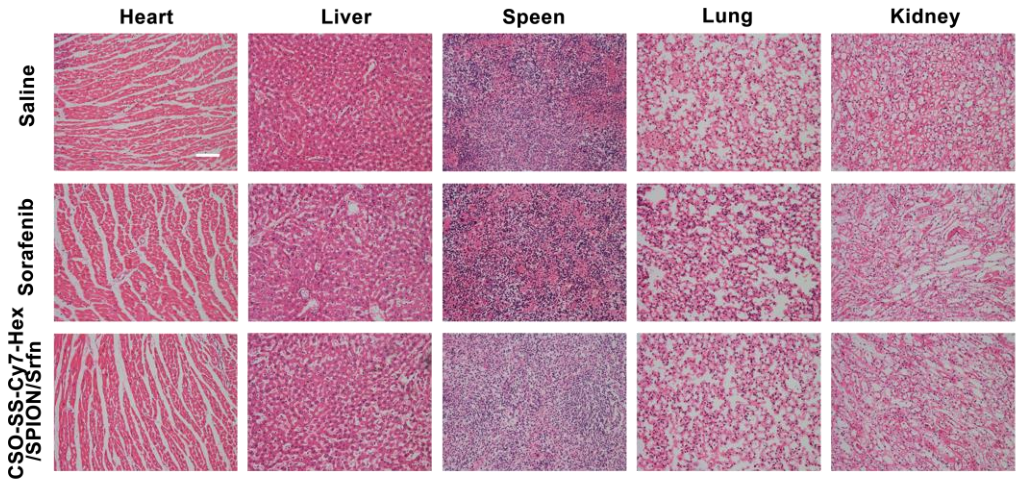

Figure S34. Biosafety of CSO-SS-Cy7-Hex/SPION/Srfn complex self-assemblies *in vivo*. H&E staining of female rat organs (hearts, livers, spleens, lungs, and kidneys) at the end of experiments. (scale bar: 200 μm).
